# Wnt Signaling in the Breast: From Development to Disease

**DOI:** 10.3389/fcell.2022.884467

**Published:** 2022-05-18

**Authors:** Willy Antoni Abreu de Oliveira, Youssef El Laithy, Alejandra Bruna, Daniela Annibali, Frederic Lluis

**Affiliations:** ^1^ Department of Development and Regeneration, Stem Cell Institute, Katholieke Universiteit (KU) Leuven, Leuven, Belgium; ^2^ Centre for Paediatric Oncology Experimental Medicine, Centre for Cancer Evolution, Molecular Pathology Division, London, United Kingdom; ^3^ Department of Oncology, Gynecological Oncology Laboratory, Leuven Cancer Institute (LKI), KU Leuven, Leuven, Belgium; ^4^ Division of Oncogenomics, Oncode Institute, The Netherlands Cancer Institute, Amsterdam, Netherlands

**Keywords:** Wnt, breast, development, cancer, signaling

## Abstract

The Wnt cascade is a primordial developmental signaling pathway that plays a myriad of essential functions throughout development and adult homeostasis in virtually all animal species. Aberrant Wnt activity is implicated in embryonic and tissue morphogenesis defects, and several diseases, most notably cancer. The role of Wnt signaling in mammary gland development and breast cancer initiation, maintenance, and progression is far from being completely understood and is rather shrouded in controversy. In this review, we dissect the fundamental role of Wnt signaling in mammary gland development and adult homeostasis and explore how defects in its tightly regulated and intricated molecular network are interlinked with cancer, with a focus on the breast.

## Introduction

Deregulation of developmental signaling pathways is common in many cancer types, a feature that underpins the similarities between embryonic development and tumorigenesis (Manzo, 2019). The Wnt signaling pathway is critical during embryonic development and crucial for adult tissue homeostasis in all animal species. In addition, its aberrant activity is implicated in the tumorigenesis of several cancer types, including breast cancer (Yu et al., 2016; Pohl et al., 2017).

The wingless (wg) gene was discovered in 1973 in *Drosophila melanogaster* by RP. Sharma, when performing mutagenesis screening of visual phenotypes. Subsequent studies identified several wingless-related factors that function as mediators of patterning during embryonic development (Nüsslein-Volhard and Wieschaus, 1980; Zhan et al., 2017).

Wnt was first associated with tumorigenesis with the discovery that overexpression of *int1* (*Wnt1*), then found to be an ortholog of *wg*, generated mammary hyperplasia and breast tumors in mice (Nusse et al., 1984; Rijsewijk et al., 1987; Tsukamoto et al., 1988).

Later, mutations in the adenomatous polyposis coli (APC) gene were discovered to be one cause of hereditary colon cancer. Soon after, the *APC* gene product would be found to be an essential regulator of the intracellular Wnt cascade, leading to the establishment of constitutive Wnt activation as a key oncogenic driver in *APC* ^*−/−*^ colon carcinomas (Kinzler et al., 1991; Nishisho et al., 1991; Rubinfeld et al., 1993; Su et al., 1993; Korinek et al., 1997).

Since the discovery of *WNT1,* the molecular complexity of the Wnt signaling cascade was progressively resolved, leading to the discovery of many components of this intricate pathway. Moreover, the intracellular responses triggered by Wnt ligands have been shown to branch into β-catenin-dependent signaling (canonical Wnt pathway) and -independent signaling (non-canonical Wnt pathway).

### Wnt Ligands, Receptors, and Natural Antagonists

Both branches of Wnt signaling are initiated by Wnt ligands (homologs of *wg* and *int1*). These growth factors are conserved throughout the animal kingdom and constitute a family of 19 known Wnt ligands in mammals, encoding for secreted glycoproteins whose function is to activate one or both branches of Wnt signaling in a paracrine or autocrine manner (Mikels and Nusse, 2006).

**FIGURE 1:**
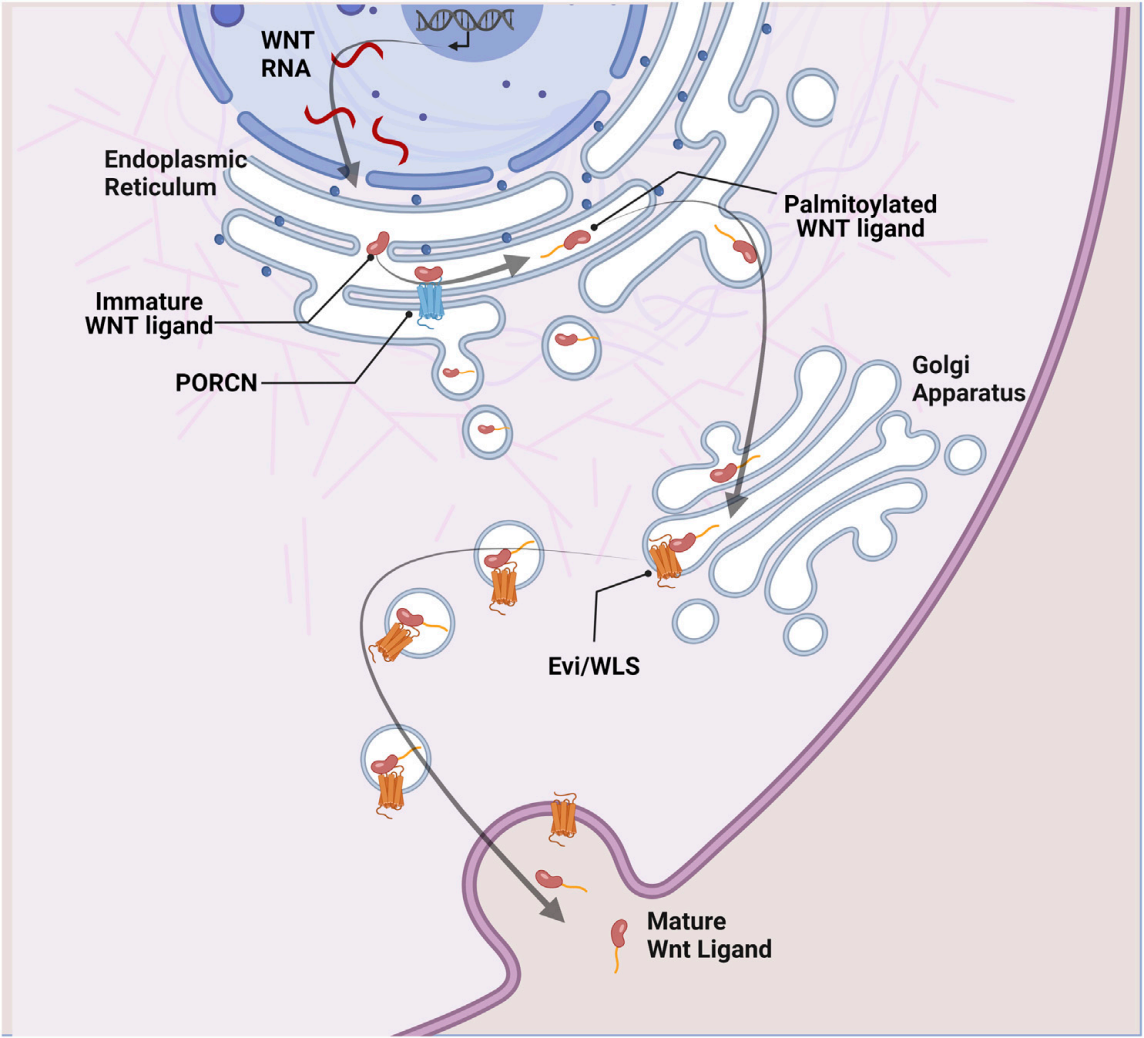
Wnt ligand secretion. In Wnt secreting cells, immature Wnt proteins are palmitoylated in the endoplasmic reticulum by the porcupine acyl transferase (PORCN). This is an essential step in the Wnt secretory pathway. Lipid-modified Wnt ligands are then transported in secretory vesicles to the plasma membrane with the help of the transmembrane protein Evi/WLS.

Before secretion, Wnt ligands undergo glycosylation in the endoplasmic reticulum. Subsequently, the Porcupine O-acyltransferase (PORCN) adds a palmitoyl group before binding Evenness interrupted WNTless (Evi/WLS) to be shuttled to the plasma membrane via Golgi apparatus. PORCN acts as a rate-limiting enzyme in the Wnt secretory pathway as acylation of Wnt ligands is essential for their secretion (Coudreuse and Korswagen, 2007; Buechling et al., 2011; Nusse and Clevers, 2017) (Figure 1).

Secreted Wnt ligands directly interact with receptor complexes in receiving cells to activate canonical or non-canonical Wnt signaling. Receptor complexes, consisting of one of 10 members of the Frizzled (FZD) family of transmembrane receptors and co-receptor low-density lipoprotein receptor-related proteins 5,6 (LRP5/6), activate canonical Wnt signaling. On the other hand, receptor complexes consisting of a FZD receptor and Receptor Tyrosine Kinase-like orphan receptor 1/2 (ROR1/ROR2) or receptor-like tyrosine kinase (RYK) activate non-canonical Wnt signaling (Martin-Orozco et al., 2019).

**FIGURE 2:**
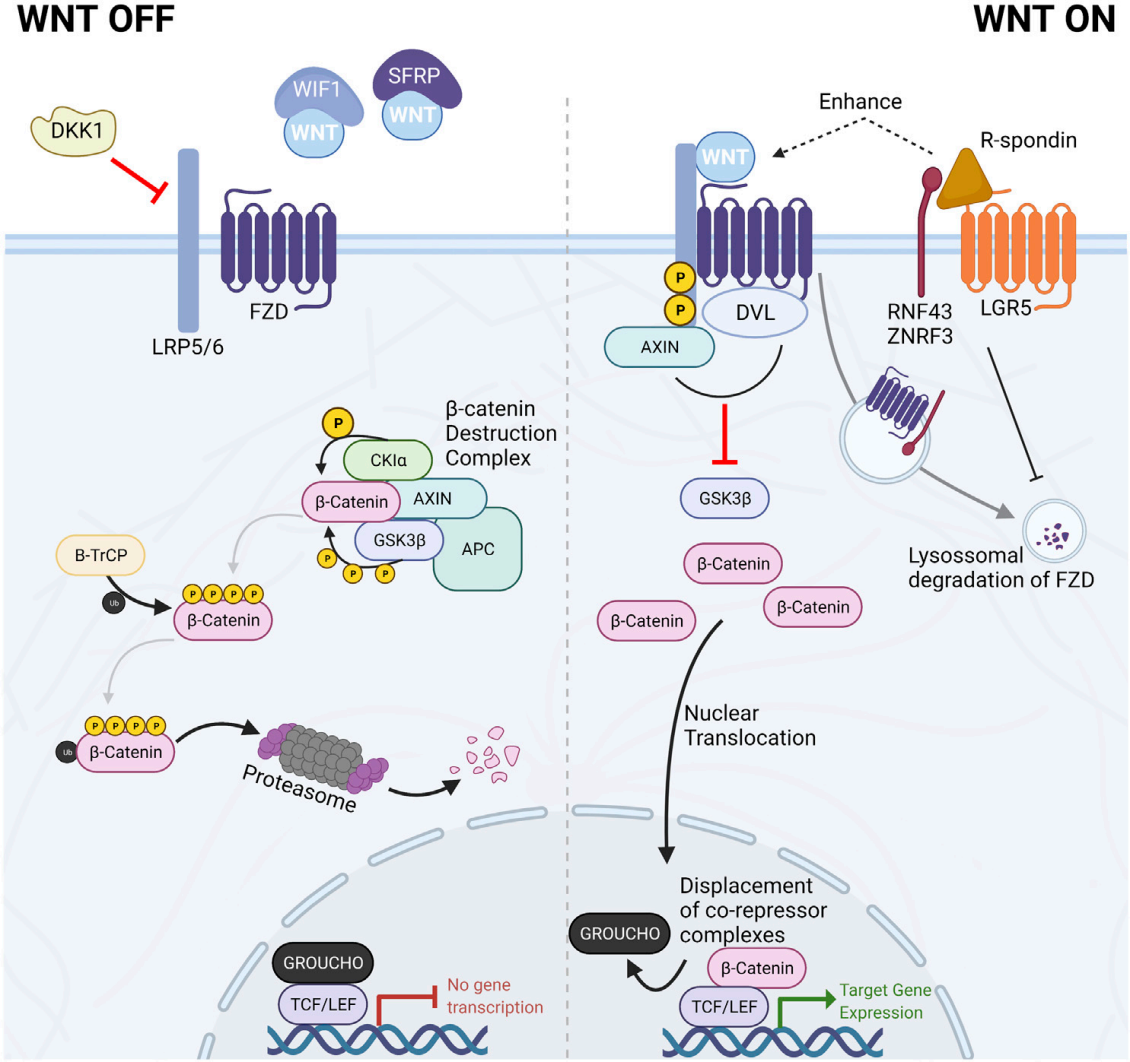
The Canonical Wnt/β-catenin signaling pathway. In the absence of Wnt ligands or in the presence of Wnt antagonists such as DKK1, WIF1, or SFRPs, the Wnt signaling pathway is kept in the *off* state by the dynamic degradation of β-catenin mediated by the β-catenin destruction complex. This multiprotein complex is composed of the scaffolding proteins AXIN1/2 and APC and the kinases CK1α and GSK3β. The two kinases sequentially phosphorylate β-catenin, targeting it for proteasomal degradation. Conversely, when Wnt ligands bind the FZD/LRP co-receptor complex, the β-catenin destruction complex is disassembled. Consequently, β-catenin accumulates in the cytoplasm and translocates to the nucleus, where it displaces co-repressors bound to TCF/LEF transcription factors, thereby initiating Wnt-target gene expression. R-spondins can amplify Wnt ligand response and increase cellular sensitivity to Wnt ligands by inhibiting the recycling of FZD receptors.

More recently, the members of the R-spondin family have emerged as important regulators and amplifiers of canonical Wnt signaling. The R-spondin (RSPO) family consists of four secreted proteins that contribute to Wnt signaling activation by synergizing with Wnt ligands. They act by binding members of the leucine-rich repeat-containing G-protein coupled receptors (LGR4-6), consequently inhibiting transmembrane E3 ubiquitin ligases, Rnf43 and Znrf3, involved in the recycling of FZD receptors (de Lau et al., 2014). These transmembrane E3-ubiquitin ligases are target genes of Wnt/β-catenin and constitute part of the negative-feedback loop of the canonical Wnt pathway. The reduced recycling of FZD receptors enhances sensitivity to secreted Wnt ligands and potentiates their effect **(** Figure 2) (de Lau et al., 2014).

In addition to secreted agonists, several classes of natural antagonists exert their regulatory effects on Wnt signaling activity. The carboxylesterase Notum has been shown to remove the palmitoyl group from Wnt ligands in *Drosophila,* thereby inhibiting their interaction with the FZD Wnt binding domain (Kakugawa et al., 2015; Zhang et al., 2015). In addition to Notum, the secreted family of Dickkopf (DKK) proteins antagonize Wnt by inhibiting FZD-LRP5/6 dimerization, thereby impeding canonical Wnt signal transduction (Cruciat and Niehrs, 2013). The secreted FZD-related family of proteins (sFRPs) and Wnt inhibitory protein (WIF) directly bind Wnt ligands in the extracellular space to inhibit their activity (Cruciat and Niehrs, 2013).

### Canonical Wnt Signaling (β-catenin Dependent Wnt Signaling)

Canonical Wnt signaling is the most studied and best understood Wnt pathway branch. Its activity converges on the central role of β-catenin as it relays Wnt-ligand mediated activation of the intracellular signaling cascade and the downstream effectors and regulators of Wnt-dependent gene expression (Figure 2).

In the absence of Wnt ligands (Figure 2—WNT OFF), the canonical Wnt signaling pathway is inactive due to the continuous degradation of β-catenin. β-catenin is sequentially phosphorylated and targeted for proteasomal degradation by members of the β-catenin destruction complex. This multiprotein complex is constituted by two scaffolding proteins [the tumor suppressors APC and axis inhibition protein (AXIN1 or AXIN2)], casein kinase 1 alpha (CK1α), glycogen synthase kinase 3β (GSK3β), and the E3-ubiquitin ligase β-TrCP. The sequential phosphorylation by CK1α at Ser45 and GSK3β at Thr41, Ser37, and Ser33, prime β-catenin for ubiquitination and consequent degradation. In this manner, cytoplasmic levels of β-catenin are kept low, impeding further signal transduction (Nusse and Clevers, 2017).

When Wnt ligands bind to the FZD/LRP receptor complex (Figure 2—WNT ON), Dishevelled (DVL) recruits AXIN disassembling the β-catenin destruction complex leading to inhibition of GSK3β. Consequently, newly synthesized β-catenin starts accumulating in the cytoplasm and becomes available for nuclear translocation. In the nucleus, β-catenin binds to the T cell factor/lymphoid enhancer factor (TCF/LEF) family of transcription factors, thereby eliciting changes in transcriptional regulation of Wnt target genes (Nusse and Clevers, 2017). The four members of the TCF/LEF transcription factors family (TCF7, LEF1, TCF7L1, and TCF7L2) are constitutively bound to DNA. However, when Wnt signaling is inactive, TCF/LEF transcription factors are bound by members of the Groucho family to mediate transcriptional repression of target genes. On the other hand, when Wnt signaling is active and β-catenin enters the nucleus, Groucho factors are displaced, and target genes become transcriptionally active (Cruciat and Niehrs, 2013; Nusse and Clevers, 2017). Wnt target genes are cell-type-, tissue- and developmental stage-specific; however, activation of canonical Wnt pathway also induces a negative feedback loop promoting expression of some destruction complex components such as *AXIN2* that is paradoxically frequently used as a generic target indicator of canonical Wnt activity (Cruciat and Niehrs, 2013; Nusse and Clevers, 2017).

### Non-Canonical Wnt Signaling (β-Catenin Independent Wnt Signaling)

Several β-catenin-independent, Wnt activated signaling cascades have been discovered. Among them, the Wnt/planar cell polarity (Wnt/PCP) and the Wnt/Ca^2+^ pathways are the most well-known. Both pathways are activated by the interaction of Wnt ligands with FZD receptors without the involvement of LRP5/6.

The Wnt/PCP pathway is involved in establishing and regulating cell polarity, motility, and migration (Luga et al., 2012; Martin-Orozco et al., 2019). Activation of Wnt/PCP is mediated by an FZD/ROR/RYK/Vang-like protein 2 (VANGL2) axis which ultimately leads to the phosphorylation of c-Jun N-terminal kinase (JNK). Phosphorylation of JNK leads to gene transcription by activator protein 1 transcription factor (AP1) (Martin-Orozco et al., 2019). This β-catenin independent pathway is also known to negatively regulate the canonical Wnt/β-catenin pathway in a DVL dependent manner (Martin-Orozco et al., 2019).

The Wnt/Ca^2+^ pathway is critical in regulating cell adhesion, migration, and embryonic development (Martin-Orozco et al., 2019). It is activated upon Wnt ligand interaction with FZD receptors and ROR/RYK. Subsequently, DVL is recruited to the receptor complex in the cell membrane’s inner leaflet, leading to phospholipase C (PLC) activation and downstream release of intracellular calcium ions. Intracellular calcium fluxes provoke the activation of calcium-dependent kinases such as calpain 1 and calcineurin. In turn, these activate transcription through the activity of nuclear factor activated T-cell (NFAT) and nuclear factor kappa B (NFκB) transcription factors (Martin-Orozco et al., 2019).

Although the role of the non-canonical Wnt pathway in mammary development and in breast disease is starting to be elucidated (see below), in this review, we mainly focus on the canonical Wnt signaling cascade due to the clearer general understanding of its underlying biology in health and disease and the high frequency of aberrant canonical Wnt activity in breast cancer.

## Wnt Signaling in Mammary Gland Development and Mammary Stem Cells

The mammary gland is a complex network of branched epithelial lobules and tubes which produce, collect, and transport milk to the nipple (Medina, 1996). This ductular-lobular structure consists of a bi-layered epithelium; the inner layer comprises luminal epithelial cells lining the lumen of the ducts and lobules, while the outer enveloping layer is made of basal myoepithelial cells subjacent to the basement membrane (Pandya and Moore, 2011).

Despite major differences between species (see below), our understanding of the molecular and histological processes underlying mammary gland development mainly stems from studies in model organisms, particularly murine models due to the limited availability of human pre-natal biological samples. From numerous studies in mice laboratory models, we know the very first morphogenic events during murine mammary gland development occur during embryonic day 10 with the appearance of the bilateral mammary lines between the limb buds. (Pandya and Moore, 2011).

**FIGURE 3:**
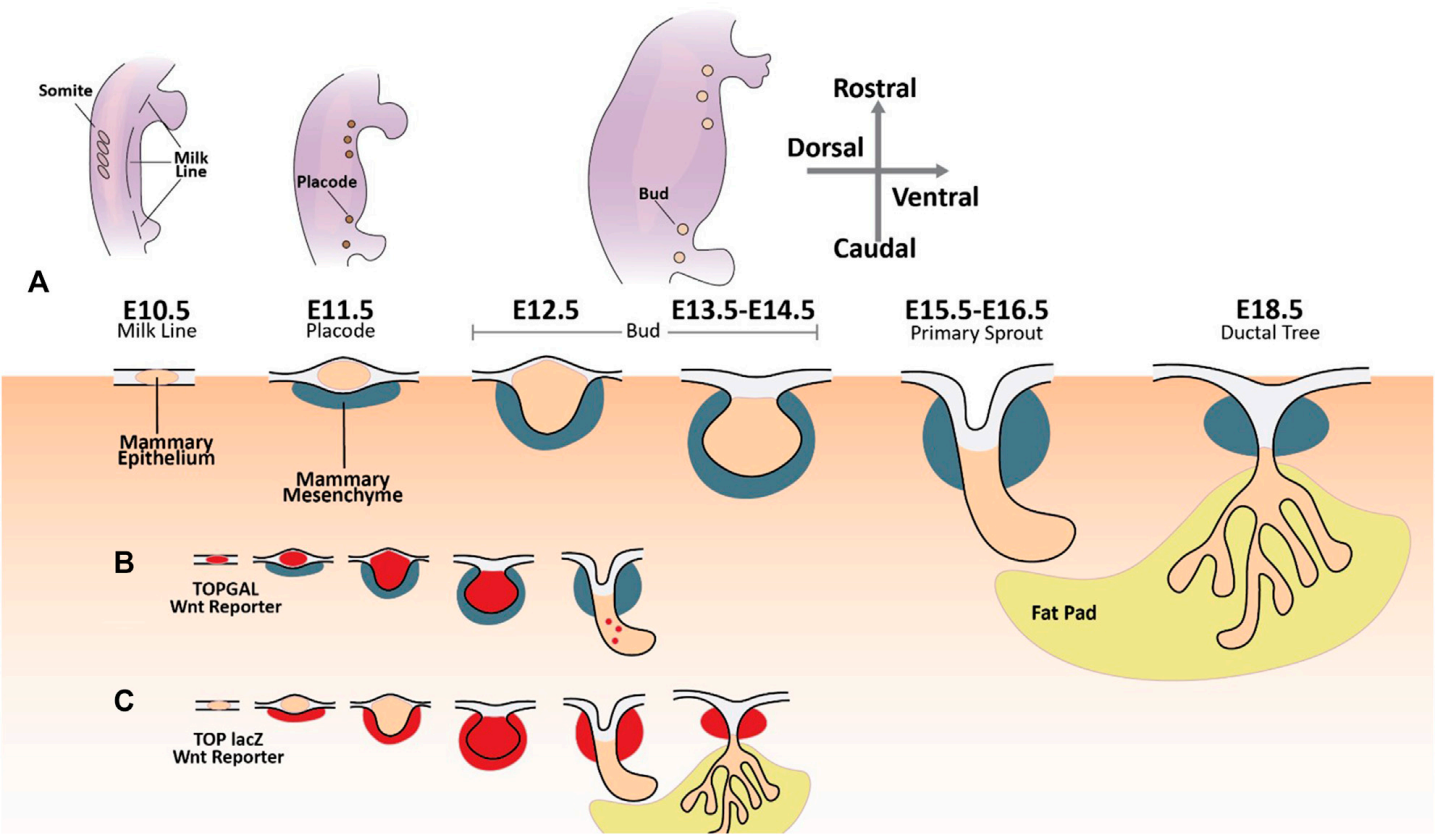
The developing mammary gland and Wnt signaling activity. **(A)** The first morphogenic event in murine mammary gland embryonic development is the specification of the mammary lines along the anterior-posterior axis of each flank around embryonic day 10.5. On embryonic day 11.5, five pairs of placodes composed of condensed mammary epithelial cells arise from the mammary line. These primordial structures undergo a series of morphological changes until a rudimentary mammary ductal tree is formed at E18.5. **(B)** TOPGAL Wnt reporter expression during embryonic mammary gland development (Wnt active areas in red) (Chu et al., 2004). **(C)** TOP lacZ Wnt reporter expression during embryonic mammary gland development (Wnt active areas in red) (Boras-Granic et al., 2006; Boras-Granic and Wysolmerski, 2008).

Five pairs of mammary placodes arise from ectodermal cells in the mammary lines at E11 (Figure 3A). By E13.5, the mammary placodes invaginate, generating mammary buds of epithelial cells surrounded by specialized mammary mesenchyme. Epithelial cells now committed to the mammary fate proliferate and invade the underlying mammary fat pad by E16.5. The mammary epithelial buds then branch out, forming a rudimentary mammary ductal tree comprised of up to twenty branches (Pandya and Moore, 2011). When females reach puberty, circulating growth hormones and estrogen drive further remodelling and maturation of the mammary gland. During this stage of development, the distal portion of each duct becomes enlarged, forming terminal end buds. These structures contain highly proliferative cell populations that drive the elongation and bifurcation of the ducts, supporting the branching of the mammary tree as it colonizes the mammary fatty stroma. But it is not until pregnancy that fully differentiated alveolar mammary epithelial milk-producing cells finally arise (Pandya and Moore, 2011).

The Wnt signaling pathway is tied to mammary gland development from very early on. Transcriptional activity of the canonical Wnt signaling pathway can be detected along the mammary lines at their inception during E10.5 using transgenic TOPGAL Wnt reporter mice and remains active until bud formation at E13.5 (Figure 3B) (Chu et al., 2004). *Wnt10b* mRNA is detected along the mammary line at E11.5 (Veltmaat et al., 2004). Moreover, embryos cultured with exogenous Wnt activators (e.g., LiCl) or Wnt ligands (e.g., Wnt3a) show accelerated and ectopic mammary placode formation with rich *Wnt10b* expression (Chu et al., 2004).

Furthermore, mammary placode formation is blocked upon transgenic expression of the Wnt inhibitor DKK1 (Chu et al., 2004). As the mammary buds arise from the placodes and commence their outgrowth and duct formation, Wnt signaling is progressively reduced (Chu et al., 2004). Apart from the early mammary epithelia, studies using a second Wnt reporter mouse line (TOP-LacZ) demonstrated that transcriptional Wnt activity is present in the mammary mesenchyme as early as E11 (Figure 3C) (Boras-Granic et al., 2006; Boras-Granic and Wysolmerski, 2008). Mammary epithelium becomes LacZ positive around E13 when bud formation occurs, but Wnt activation subsides rapidly in this compartment as primary sprouting starts. On the other hand, LacZ activity remains in the mesenchymal compartment under the nipple during duct formation (Boras-Granic et al., 2006; Boras-Granic and Wysolmerski, 2008). Wnt activation has also been reported in the mesenchymal cells underlying the arising placodes as early as E11 with sustained expression until E13 (Boras-Granic et al., 2006).

Genetic ablation of *Lef1* has shown its requirement for the formation of placodes 2 and 3, specifically, suggesting that Lef1 activity is necessary following mammary line formation (Boras-Granic et al., 2006). The expression of this transcription factor is tightly regulated and time-dependant. It is first expressed in mammary epithelial cells at E11-E12. However, after mammary bud formation (E14-E15), Lef1 expression is exclusive to mesenchymal cells (Foley et al., 2001). *Lef1* expression has been shown to be under the control of parathyroid hormone-related protein (PTHrP). Overexpression of PTHrP leads to improper differentiation of the ventral epidermis and ectopic expression of *Lef1* and β-catenin, together with mesenchymal markers. Conversely, ablation of PTHrP expression disrupts transcriptional Wnt reporter activity (Hiremath et al., 2012). Furthermore, deletion of β-catenin in the mammary mesenchyme severely disrupted mammary bud formation and establishment of sexual dimorphism, highlighting the pivotal importance of mesenchymal-specific Wnt activity in the correct progression of developmental processes towards the epithelial fate (Hiremath et al., 2012).

The most radical changes during mammary gland development occur postnatally. Both canonical and non-canonical Wnt cascades are involved in ductal growth during puberty and adult mammary epithelium maintenance. The pubertal outgrowth of mammary gland ducts is driven by the proliferative Terminal End Buds (Paine and Lewis, 2017). These structures are enriched for the expression of several Wnt ligands, most notably Wnt5a and Wnt7b, while the adjacent stroma is enriched for Wnt2 (Buhler et al., 1993; Kouros-Mehr and Werb, 2006).

More recently, Ror2, a non-canonical Wnt ligand-receptor, was shown to mediate Wnt5a-dependent antagonism of Wnt/β-catenin signaling in mammary epithelial cells, thereby inhibiting branching and outgrowth of mammary ducts. Significantly, depletion of Ror2 expression was shown to enhance mammary branching *in vivo* (Roarty et al., 2015).

Understanding the biology of the mammary stem cell (MaSCs) compartment has great implications for both development and disease, particularly in cancer. Roarty and colleagues described an intricate interplay between β-catenin dependent and independent signaling in the control of MaSC self-renewal and differentiation during mammary gland development and branching. Their work proposes a model in which β-catenin dependent signaling maintains the self-renewal of MaSCs in the terminal end bud (the leading edge of mammary gland branch extension). Conversely, Ror2 expression in the duct, being formed in the wake of the terminal end bud, leads to the differentiation of mammary epithelial cells within the basal and luminal breast lineage (Roarty et al., 2015).

Badders and colleagues established Lrp5 as the first biomarker of mouse mammary stem cells and demonstrated that Lrp5^+^ cells in mammary epithelial cell cultures had significant stem cell activity (Badders et al., 2009). Moreover, adult mammary gland cells with active Wnt/β-catenin are enriched for MaSCs, and Wnt ligands have been shown to function as self-renewal promoting factors in the adult murine mammary gland (Zeng and Nusse, 2010).

Following the explosion of scientific interest in stem cell biology, a large number of studies were conducted to identify and characterize in detail the murine mammary gland stem cells and hierarchical differentiation (Stingl et al., 2006; Ginestier et al., 2007; Korkaya et al., 2009; Inman et al., 2015). Albeit the presence of many apparent discrepancies potentially due to the use of different experimental models and analytical tools, most reports support the existence of unipotent basal and luminal stem cell pools involved in the development, homeostasis, and remodeling of the postnatal mammary gland. Notwithstanding, extensive experimental and omics evidence also supports the presence of bipotent stem cells. These are long-lived progenitor cells with considerable expansion capacity and the function of regulating ductal homeostasis and architecture (Rios et al., 2014; Chen et al., 2019). Moreover, the expression of LGR5, an RSPO receptor and, consequently, a canonical Wnt signaling-related receptor, identifies a population of fetal mammary stem cells with bipotent differentiation capacity, in agreement with the role of canonical Wnt signaling (Trejo et al., 2017).

Despite the large amounts of evidence supporting the involvement of Wnt signaling in the regulation of murine mammary gland development and homeostasis, particular attention should be taken when translating these findings to the human breast, given that significant structural, histological, and molecular differences exist between the human and murine mammary glands (Dontu and Ince, 2015). Specifically, the murine mammary ductal tree is less complex than the human counterpart, and the murine stroma contains more significant amounts of adipocytes. In contrast, the human breast possesses a denser, more specialized stroma with higher fibroblast and collagen content (Dontu and Ince, 2015). These structural and histological differences likely underpin disparate mechanical and signaling features in the two species. In addition, the limited access to healthy human tissue significantly restricts the advancement of comparative studies (van Schie and van Amerongen, 2020). Notwithstanding, transcriptome analysis of putative human ALDH^+^, CD44^+^/CD24^-^ breast stem cells suggests the importance of autocrine Wnt signaling for their maintenance, as expression levels of *WNT2* and R-spondin 3 (*RSPO3*) are elevated in this population (Colacino et al., 2018).

## Breast Neoplastic Disease

Breast cancer is the number one neoplastic cause of death in women and the most frequently diagnosed malignancy. Globally, in 2020, breast cancer accounted for almost 12% of all neoplastic disorders, having been diagnosed in over two million women (Sung et al., 2021). In recent years, new technological advances enabled molecular profiling of breast cancer at unprecedented resolution solidifying heterogeneity as breast cancer’s most fundamental feature and warranting the development of more robust molecular prognostic signatures and therapeutic options. Due to the significant prognostic and predictive consequence of such diversity, researchers around the world turned their attention towards refining the well accepted and highly annotated histopathological classification based on the expression of the well-known breast cancer molecular biomarkers Estrogen receptor (ER), Progesterone Receptor (PR), and Human Epidermal Growth Factor Receptor 2 (HER2) which guide treatment choices. For example, an estrogen receptor-positive (ER+) tumor dictated the use of anti-estrogen treatment (such as Tamoxifen), one of the first of its kind and most effective targeted therapy in the history of cancer medicine (Loi et al., 2010). Therefore, the first and most frequently used molecular classification was based on the expression of these three breast cancer receptors, which encompasses a particularly aggressive subtype defined by the lack of ER, PR, and HER2 expression called the triple-negative breast cancer subtype (TNBC).

The development of molecular classifiers was linked to the advent of new technological advances such as gene expression profiling. Notably, in the early 2000s, extensive work by Perou, Sørlie, and colleagues classified breast cancers into five main molecular subclasses, based on gene expression profiles called the “intrinsic subtypes”: Luminal A and Luminal B, human epidermal growth factor type 2 enhanced (HER2), Basal-like (BLBC), and Normal-like (NLBC) breast cancers (Perou et al., 2000; Sørlie et al., 2001).

Following this work, further classifications using gene signatures have been proposed. Using 706 cDNA probe elements, Sotiriou et al. identified six breast carcinomas groups: three luminal-like, one HER2-like, and two basal-like subtypes (Sotiriou et al., 2003). Lehmann et al. have further subdivided triple-negative tumors into six stable groups—2 basal-like (BL1 and BL2), one immunomodulatory (IM), one mesenchymal (M), one mesenchymal stem-like (MSL), and one luminal androgen receptor (LAR) subtype (Lehmann et al., 2011).

With the emergence of powerful technologies such as Next Generation Sequencing (NGS) platforms, more studies have provided extensive and detailed insight into breast cancer’s heterogeneous genomic and transcriptomic architecture for the discovery of new novel subtypes (Dai et al., 2015). A study of 2000 breast tumors performed by Curtis et al. paved the way in integrating data at multiple levels (genomic and transcriptomic level). This study revealed a refined breast cancer molecular taxonomy by introducing ten integrative clusters named IntClust 1–10 derived from the impact of somatic copy number aberrations (CNAs) on the transcriptome (Curtis et al., 2012).

In parallel, The Cancer Genome Atlas Network (TCGA) investigated breast cancer subtypes by incorporating data from multiple platforms, including, exosome sequencing, mRNA arrays, DNA methylation, genomic DNA copy number arrays, and more. They concluded that diverse genetic and epigenetic alterations converge phenotypically into four major breast tumor subgroups (Luminal A, Luminal B, HER2 positive, and Triple-negative) as previously identified by Sørlie et al. (Cancer Genome Atlas Network, 2012). Despite the variability in naming and the number of categories grouped by different studies, the classical subtyping by Sørlie et al. still provides a highly significant classification method from which we will use for this review.

### Hormone Receptor-Positive Breast Cancers

Roughly 70% of breast cancers are intrinsically dependent on steroid hormone signaling. The Luminal A and Luminal B breast cancer subtypes are positive for ER and/or PR (Perou et al., 2000; Feng et al., 2018; Harbeck et al., 2019; Waks and Winer, 2019). Patients diagnosed with early, stage I, luminal breast cancers generally have an excellent prognosis, with over 99% of patients achieving 5-year breast cancer-specific survival. These tumours are driven by estrogen-dependent oncogenic events (Waks and Winer, 2019). Specifically, ERα facilitates the proliferation of tumor cells by inducing Cyclin D1 activity, an essential mediator of cell cycle progression, which governs the transition from the G1 to S phase (Cicatiello et al., 2004). Significantly, one of the few and most successful standards of care precision medicine approaches based on matching molecular targets with treatment choices is endocrine therapy in hormone receptor-positive breast cancer patients.

Fifteen to twenty percent of breast cancers overexpress the HER2 oncogene. HER2 is an orphan receptor whose activation leads to downstream induction of several pro-proliferative and pro-survival signaling cascades such as the mitogen-activated protein kinase (MAPK), extracellular signal-regulated kinase (ERK), and the phosphatidylinositol 4,5-biphosphate 3-kinase (PI3K) (Burgess, 2008; Feng et al., 2018). HER2 enriched cancers can either be positive or negative for ER and PR and are more aggressive and less tractable than luminal tumors. Like the ER in luminal cancers HER2 is a druggable target, with HER2-targeted monoclonal antibodies such as trastuzumab being utilized as one of the main therapeutic weapons (Feng et al., 2018; Loibl et al., 2021).

### Triple-Negative Breast Cancers

TNBC encompasses a heterogeneous group of breast cancers with distinct clinical characteristics, transcriptomic and genomic features, and unique histopathological differences marked by the lack of expression of ER, PR, and HER2 (Borri and Granaglia, 2021). TNBC remains a subgroup with a poor prognosis due to an aggressive phenotype and lack of actionable targets (Dent et al., 2007). The current standard of care usually involves high doses of chemotherapy followed by surgery, but unfortunately, the prognosis is dismal, especially for patients with no pathological complete response (pCR) (Wahba and El-Hadaad, 2015; Bergin and Loi, 2019; Waks and Winer, 2019).

The heterogeneous transcriptomic landscape of TNBCs was resolved in-depth in two landmark publications by Lehman and colleagues in 2011 and 2016 (Lehmann et al., 2011, 2016). By analyzing gene expression data from 21 datasets in the 2016 study, TNBCs were clustered into four subtypes: basal-like type 1, basal-like type 2, mesenchymal, and luminal androgen receptor (Lehmann et al., 2016). The basal-like subtype 1 is characterized by high proliferation coupled with enhanced DNA damage response (DDR) pathways (Hubalek et al., 2017). The basal-like type 2 is enriched in growth factor signaling pathway activity, including epidermal growth factor (EGF), nerve growth factor (NGF), and the Wnt signaling pathway (Hubalek et al., 2017). The mesenchymal and mesenchymal stem-like subtypes have enhanced cell motility, differentiation, and growth pathways, including platelet-derived growth factor (PDGF) (Hubalek et al., 2017). Finally, the luminal androgen receptor subtype is the more dissimilar among TNBCs. It is particularly enriched in steroid hormone synthesis and signaling pathways, displaying androgen receptor and ER signaling, despite being ERα negative by immunohistochemistry (IHC) (Hubalek et al., 2017).

The stratification of TNBCs into relevant molecular and clinical subtypes could prove helpful in improving treatment prediction and prognostic algorithms. Several studies analysed the response of TNBC subtypes to neoadjuvant chemotherapy and found significant differences. For instance, a retrospective study by Masuda and colleagues revealed vast differences, with basal-like type 1 TNBCs achieving pCR in 52% of cases and basal-like type 2, androgen receptor and mesenchymal TNBCs achieving pCR rates of 0, 10, and 23%, respectively (Masuda et al., 2013). However, despite the striking differences in clinical behavior and biological features of these putative TNBC subtypes, their use has not yet been translated into clinical practice (Marra et al., 2020).

A key characteristic of TNBCs is their insufficient DNA damage repair capacity and increased genomic instability (Denkert et al., 2017). Additionally, a significant proportion of TNBCs is *BRCA1* mutated—75% of women carrying germline mutations of this gene develop TNBCs (Foulkes et al., 2010). BRCA1 is critically involved in mediating the repair of double-strand DNA breaks by homologous recombination, an error-free high-fidelity DNA double-strand break repair mechanism (Park et al., 2018). Recent advances in our understanding of the DDR in breast cancer have identified new potential paths for targeted therapeutic interventions (Denkert et al., 2017; Lee et al., 2020).

## Wnt Signaling in Breast Cancer

The role of the Wnt signaling pathway is not just limited to the development and maintenance of the healthy breast and the mammary gland; it also plays an equally prominent role in breast cancer pathogenesis (Xu et al., 2020).

Deregulation and mutations in the Wnt signaling pathway have been investigated in primary human breast cancer. In contrast to colon cancer, which commonly contains gain or loss of function mutations in components of the Wnt signaling cascade, very few of such mutations have been reported in breast cancers (Pohl et al., 2017). However, upregulation of Wnt activity has been detected in a significant proportion of breast cancer, with 60% of breast cancers being characterized by high levels of β-catenin expression (Howe and Brown, 2004). In addition, elevated expression of β-catenin was correlated with high levels of the Wnt target gene *CCND1* (CyclinD1) and with poor prognosis (Howe and Brown, 2004). Furthermore, several reports demonstrate altered expression of Wnt pathway components at RNA or protein levels (Howe and Brown, 2004). For example, a reduced expression of Wnt-inhibitory factor (WIF1) was seen in 60% of invasive breast carcinomas (Wissmann et al., 2003), and *SFRP1* expression, a secreted Wnt antagonist present in breast epithelium, was shown to be lost in 80% of invasive breast carcinomas (Ugolini et al., 2001).

While the provenance of aberrant Wnt signaling levels in breast cancer seems to be veiled by uncertainty in stark contrast with other Wnt-driven cancers such as colorectal, its relationship with poor clinical outcome and poor drug-response draws parallels with such diseases. Here we focus our attention on the role of aberrant Wnt signaling in breast pathogenesis and drug response, with a particular interest in the TNBC subtype.

### Wnt Signaling and Therapy Resistance in Hormone Receptor Positive Breast Cancer

Tamoxifen (active metabolite 4-OH tamoxifen [4-OHT]), a selective estrogen receptor modulator, is the first clinically approved ER-targeted agent and the most widely used hormonal treatment for breast cancer in both pre- and post-menopausal women (Loi et al., 2010; Guan et al., 2019). Despite the success of tamoxifen therapy in reducing the annual breast cancer death rate, one-third of women treated with tamoxifen for 5 years will have recurrent disease within 15 years (Early Breast Cancer Trialists’ Collaborative Group, 2005). Tamoxifen resistance has been reported to be linked with both the canonical and noncanonical Wnt signaling pathways (Loh et al., 2013). Loh et al. (2013) demonstrated that a Tamoxifen-resistant *in vitro* model exhibited increased transcriptional levels of canonical Wnt signaling, and WNT3a supplementation further increased resistance of the parental and resistant model to Tamoxifen treatment . Moreover, SOX2 (a transcription factor essential in maintaining self-renewal and pluripotency in embryonic stem- and somatic cells) was enriched in Tamoxifen-resistant cells, correlating with an increased Wnt signaling activation (Piva et al., 2014).

Despite HER2 targeting agents, such as trastuzumab, substantially improved the prognosis of the HER2 subtype, the development of resistance and tumor recurrence remains a major concern. Possible mechanisms explaining resistance to trastuzumab include—but are not limited to—overexpression of other HER family receptors, increased expression and activity of c-Met, and loss of tumor suppressor phosphatase and tensis homolog (PTEN) (Shah and Osipo, 2016).

Additionally, cancer stem cells (CSCs), which have been hypothesized to significantly play a role in resistance to therapy (see also below), have been reported to have a strong correlation with the overexpression of HER2 in different breast cancer models (Korkaya et al., 2008; Ithimakin et al., 2013; Shah and Osipo, 2016). Korkaya et al. (2009) have demonstrated that HER2 regulates breast CSCs intrinsically through the PI3 kinase, AKT, and Wnt signaling pathways. Specifically, HER2 interacts with Wnt signaling through its downstream mediators ERK and AKT, both known to phosphorylate and inhibit GSK3β (a β-catenin inhibitor) (Yamaguchi et al., 2014). Wu et al. (2012) reported that Wnt3 was upregulated in HER2 cancer lines acquiring trastuzumab resistance *in vitro*, an upregulation that was correlated with increased activation of Wnt signaling. In line with those findings, knockdown of Wnt3 decreased EGFR expression, rescued trastuzumab resistance, and reduced invasiveness (Wu et al., 2012). Moreover, Hallett et al. demonstrated that inhibition of Wnt signaling using a small molecule antagonist (PKF118-310) in a HER2/Neu mouse model of breast cancer eradicated breast tumor-initiating cells *in vitro* and *in vivo* (Hallett et al., 2012).

### Wnt Signaling in Triple-Negative Breast Cancer

Colorectal cancer is a classic example of neoplastic disease driven by mutations of tumor-suppressor genes encoding the Wnt signaling pathway members. In colorectal cancer, loss-of-function mutations of the *APC* gene led to stabilization and accumulation of β-catenin and constitutive transcriptional activation of Wnt/β-catenin target genes through TCF/LEF:β-catenin activity (Kinzler et al., 1991; Nishisho et al., 1991; Su et al., 1993; Korinek et al., 1997). Alternatively, point mutations of the β-catenin coding gene *CTNNB1* lead to alterations in the protein’s N-terminal Ser/Thr phosphorylation sites, thereby preventing phosphorylation by GSK3β and consequent degradation in some *APC* wild-type colorectal cancers (Morin et al., 1997).

Mutations in other components of the Wnt/β-catenin signaling cascade are known oncogenic drivers of many other neoplastic disorders such as cancers of the liver, stomach, pancreas, ovary, endometrium, kidney, adrenal gland, biliary tract, pituitary, and soft tissues (Zhong and Virshup, 2020).

Interestingly, aberrant Wnt/β-catenin signaling is a feature of breast cancers, specifically TNBCs (Khramtsov et al., 2010; Xu et al., 2015; van Schie and van Amerongen, 2020). Kharamtsov and colleagues analyzed breast cancer tissue microarrays for the subcellular localization of β-catenin and showed that nuclear and cytoplasmatic levels were substantially enriched in basal-like breast cancers (Khramtsov et al., 2010). Significantly, increased β-catenin protein levels were also associated with stem cell enrichment (Khramtsov et al., 2010). However, oncogenic driver mutations of gain/loss of function in Wnt/β-catenin signaling components such as *APC, AXIN1, or CTNNB1* are surprisingly virtually inexistent in TNBCs. Alterations in *APC*, for instance, account for only 2.4% of breast cancer cases, compared to a staggering 73% of colorectal cancers (van Schie and van Amerongen, 2020). Regarding *CTNNB1,* the frequency of alterations in colorectal cancer is roughly 10-fold higher than in breast cancer (5 *vs*. 0.6%) (van Schie and van Amerongen, 2020).

**FIGURE 4:**
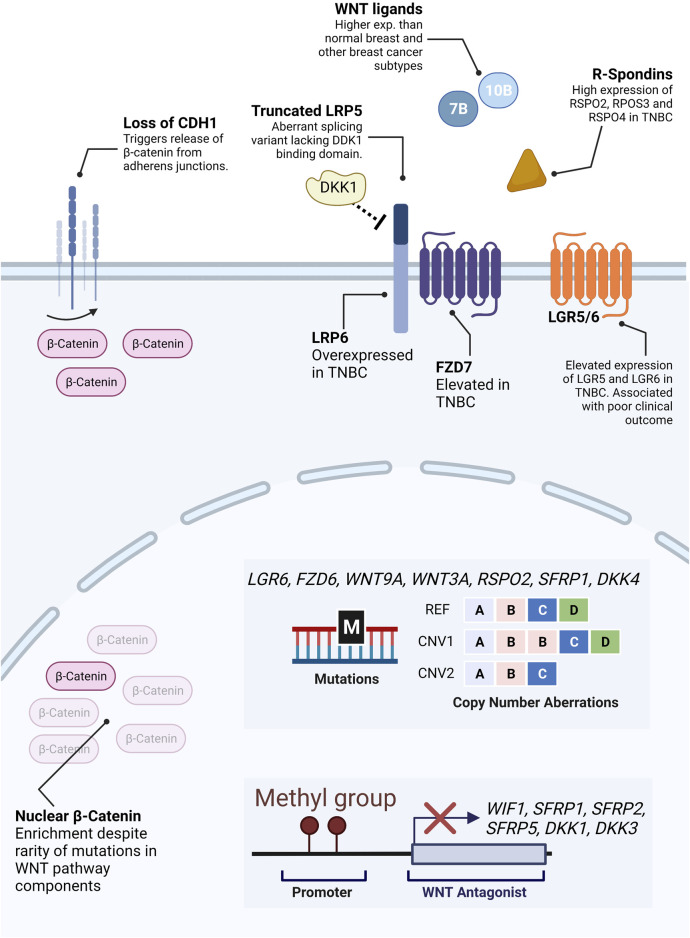
Molecular alterations in Wnt pathway components. Unlike colorectal cancer, direct alterations in β-catenin or β-catenin regulating proteins are virtually inexistent in TNBC. Notwithstanding, a significant proportion of TNBC patients display aberrant levels of β-catenin expression. While there is no clear answer to why, several possible mechanisms have been described to contribute to aberrant β-catenin levels in TNBC. These include the release of β-catenin from plasma membrane pools due to loss of CDH1, mutations or copy number aberrations (illustrative representation–REF: reference, CNV1: duplication of gene B, CNV2: deletion of gene D) in receptors and secreted Wnt agonists and antagonists, hypermethylation of promoters of genes encoding antagonists, expression of truncated antagonist receptors, etc.

The cause of such aberrant activity of the Wnt/β-catenin pathway in TNBC in the absence of known driver mutations is still not fully understood. However, over recent years, the pieces of this puzzle are being put together, one at a time (Figure 4).

Some studies suggest that elevated β-catenin levels in breast cancer could be partially due to loss of Cadherin 1 (CDH1), a frequently observed feature in advanced and invasive tumors (Prasad et al., 2008). In normal breast, β-catenin is mainly accumulated in the cell membrane, where it binds CDH1 in the adherent junctions (Hashizume et al., 1996).

Another hypothesis lies in possible changes in expression levels of upstream regulators of Wnt signaling, such as agonistic and antagonistic ligands, secreted inhibitors, and receptors (van Schie and van Amerongen, 2020). The top Wnt-related genes with genetic alterations (mutations or copy number alterations) are *LGR6, FZD6, WNT9A, WNT3A, RSPO2, SFRP1,* and *DKK4* (van Schie and van Amerongen, 2020). Alterations in these genes are comparatively more frequent in breast cancers than in colorectal neoplasms (van Schie and van Amerongen, 2020).

In addition, several studies report increased expression and/or alterations of either canonical Wnt ligands and/or members of the FZD family of Wnt receptors in breast cancer. For instance, an abnormal splicing variant of *LRP5* has been reported in breast cancer, lacking the region coding for the portion of the receptor which interacts with the Wnt antagonist DKK1(Björklund et al., 2009). In addition, *LRP6* overexpression has been detected in TNBC patients, and protein levels of the canonical WNT receptor FZD7 have been shown to be elevated in TNBCs in comparison with non-TNBCs (Yang et al., 2011; Ma et al., 2017). Interestingly, despite being the first Wnt gene associated with breast cancer tumorigenesis in mice, WNT1 is hardly found overexpressed in human breast cancers (Meyers et al., 1990). Instead, WNT10B was found to be significantly expressed specifically in TNBCs, where it is associated with larger tumor size, higher grade, and recurrence rates (Wend et al., 2013). Moreover, in 10% of breast cancers, WNT7B is expressed at levels 30-fold higher than normal breast tissue (Huguet et al., 1994).

Epigenetic regulation of Wnt-related genes is another potential cause for aberrant Wnt expression. Several studies point towards the hypermethylation of genes encoding Wnt signaling inhibitors such as *WIF1, SFRP1, SFRP2, SFRP5, DKK1,* and *DKK3* (van Schie and van Amerongen, 2020). Moreover, in 2018 Koval and Katanaev reported an overall overactivation of Wnt signaling in breast cancers and a loss of the coordination of expression of Wnt components and targets present in healthy breast tissue. The loss of this coordination, possibly through epigenetic dysregulation, likely leads to unrestricted activation of the Wnt signaling pathway and oncogenic transformation with a high degree of inter-patients’ variability (Koval and Katanaev, 2018).

Another potential driver of enriched Wnt signaling in breast cancers is the aberrant activity of secreted Wnt potentiators such as R-spondins. The primary described function of R-spondins is to potentiate Wnt signaling activity by reducing FZD receptor degradation, thereby enhancing sensitivity to extracellular Wnt ligands (Tocci et al., 2020). Several members of the R-spondin family are overexpressed in TNBC, namely RSPO2, RSPO3, and RSPO4. Accordingly, LGR5 and LGR6 are highly expressed in TNBCs and basal-like breast cancers and are associated with poor clinical outcomes and stem cell maintenance through Wnt signaling (Yang et al., 2015; Hou et al., 2018).

Developmentally conserved signaling pathways such as Wnt, Notch, and Sonic Hedgehog and their respective crosstalk, have been deemed as instrumental in mammalian development and homeostasis, as well as contributors to tumor initiation of breast cancer when dysregulated (Li et al., 2007; Kanwar et al., 2010; Wang et al., 2010). During the last years, growing evidence has emerged of the strong interaction between the Notch and Wnt pathways (Collu and Brennan, 2007; Muñoz Descalzo and Martinez Arias, 2012), suggesting both may work as a single tightly regulated module. The Notch pathway is a cell-cell interaction signaling pathway where transmembrane ligands (Jagged-1, -2, and Delta-like-1, -3, and -4) and receptors (Notch 1–4) from neighboring cells contact to promote the release of the Notch intracellular domain (NICD) which upon nuclear translocation promotes transcriptional regulation of target genes. For a deeper understanding of the Notch pathway, read (Ranganathan et al., 2011; Kovall et al., 2017). The synergism between the Wnt and Notch occurs at several levels. Wnt/β-catenin triggers the expression of Notch ligands, and Notch activity leads to the expression of Wnt genes (Muñoz Descalzo and Martinez Arias, 2012). Furthermore, several interactions between internal components of both pathways have been described, such as NICD-Axin (Hayward et al., 2006), NICD-Dishevelled (Axelrod et al., 1996; Muñoz-Descalzo et al., 2010) in *Drosophila* and even NICD-β-catenin in mammalian neural progenitor cells (Shimizu et al., 2008).

Some Wnt-Notch interactions support a dependent sequential activation of both pathways. However, other studies have proposed a negative loop whereby Notch restricts the activity of the Wnt pathway (Muñoz Descalzo and Martinez Arias, 2012), suggesting dysregulation or mutation of Notch components may have a profound effect on Wnt pathway activation and vice versa. Although the interaction between Wnt and Notch started to be studied in *Drosophila* recent studies have begun to shed light on the Wnt-Notch synergism in cancer and specifically in the breast. Retroviral expression of Wnt1 in human mammary epithelial cells (HMECs) promotes HMEC transformation and tumor formation through a Notch-dependent mechanism (Ayyanan et al., 2006; Collu and Brennan, 2007). Chemotherapy and radiotherapy increase the production of Wnt and Notch ligands in cells of the tumor stroma, thereby resistance in tumor cells (Sun et al., 2012; Zheng et al., 2017; Shen and Kang, 2018).

Interestingly, alteration and hyperactivation of Notch signaling has the potential to cause breast cancer, and expression of the Notch 1 receptor is associated with poor prognosis (Imatani and Callahan, 2000; Hu et al., 2006). Specifically, gain-of-function mutations in Notch receptors NOTCH1 and NOTCH2, as well as increased expression of NOTCH1 and NOTCH3, are found in breast cancer (Lee et al., 2008; Yamaguchi et al., 2008; Robinson et al., 2011; Choy et al., 2017). Whether any of the Notch mutations have a direct effect on the Wnt pathway is a matter still under investigation. However, it might provide a possible new source of deregulated Wnt activity (independent of Wnt mutations) in breast cancer.

## Wnt Signaling and TNBC Drug Resistance

The development of resistance to chemotherapy treatment in TNBC is an intriguing phenomenon, considering the extensive initial response of these tumors to neoadjuvant chemotherapy. As mentioned above, several studies have established that chemotherapy resistance in TNBC arises from a progressive adaptation of cancer cell populations to aggressive cytotoxic treatments rather than by the selection of subpopulations harboring pre-existing resistance-enabling mutations (Kim et al., 2018; Echeverria et al., 2019).

For decades, cancer researchers focused on pinpointing oncogenic drivers and resistance-enabling mutations. However, it is becoming clear that both oncogenesis and resistance to treatment are dynamic and progressive processes. In the case of resistance, increasing evidence suggests the critical role of a combination of cellular alterations such as transcriptional reprogramming, epigenetic aberrations, and signaling networks’ rewiring.

One good example of the role of Wnt signaling in mediating the acquisition of drug resistance is in *BRCA*-mutated epithelial ovarian cancers. These neoplasms display excellent initial response rates to PARP inhibitors but often develop resistance to treatment. Fukumoto and colleagues have recently shown that an interesting and novel epigenetic modification of FZD10 mRNA can arise during PARP inhibitor treatment in this cancer type. Succinctly, N^6^-methyladenosine-modified *FZD10* transcripts lead to increased mRNA stability and Wnt/β-catenin activity. Importantly, depletion of *FZD10* reinstated PARPi sensitivity while reinforcing N^6^-methyladenosine enhanced PARPi inhibition (Fukumoto et al., 2019). Importantly, these mechanisms could easily mediate chemotherapy resistance by improving DDR against DNA damaging chemotherapeutic agents or radiotherapy (Zhong and Virshup, 2020).

Interestingly, induction of Wnt signaling during drug treatment and radiotherapy has been reported, at least *in vitro,* in several cancer types, including breast cancer, where it correlates with increased resistance (Watson et al., 2010; Gao et al., 2013; Nagano et al., 2013; Emons et al., 2017; Wang et al., 2020). However, the molecular mechanisms leading to this activation and whether upstream ligands are involved in Wnt-mediated chemo-adaptation are poorly understood (Zhong and Virshup, 2020).

The Wnt pathway is known to crosstalk with other signaling cascades in health and disease (Itasaki and Hoppler, 2010; Collu et al., 2014; Morris and Huang, 2016). Therefore, it is likely that such interactions in cancer can be disrupted or exploited to benefit the survival of cancer cells. Recently, overexpressed RAS-ERK, β-catenin, and EGFR are positively correlated in TNBC, contributing to stemness and drug resistance (Ryu et al., 2020). Like Wnt/β-catenin signaling, mutations in EGFR and RAS-ERK are extremely rare in TNBC despite the high frequency of patients with incongruently high levels of activity of these signaling cascades (Ryu et al., 2020).

Wnt signaling has been shown to contribute to drug resistance in several cancers, including neoplasms of the breast. For instance, expression of the multi-drug resistance gene 1 (MDR1) is directly regulated by WNT/β-catenin signaling at different levels. FZD1 and Pygopus Family PHD Finger 2 (PYGO2), a recently discovered co-activator of Wnt/β-catenin -dependent transcription, were found overexpressed in chemotherapy-resistant breast cancer cell lines and to be essential for the maintenance of MDR1 expression. Interference and silencing of FZD1, PYGO2 and/or β-catenin ultimately restored sensitivity to doxorubicin (Zhang et al., 2012, 2016).

In addition to directly regulating the expression of drug-resistance mediating efflux pumps, aberrant Wnt signaling seems to be involved in controlling tumor immune suppression by regulating the exclusion of infiltrating lymphocytes from the tumor microenvironment (Li et al., 2019; Martin-Orozco et al., 2019). As mentioned before, high levels of lymphocyte infiltration in TNBC are correlated with improved clinical outcomes and chemotherapy response.

Wnt signaling has also been shown to regulate PD-L1 expression in TNBC cells, which plays a critical role in mediating tumor immune evasion (Castagnoli et al., 2019). High PD-L1 levels correlate with transcriptional upregulation of Wnt signaling and stem cell markers, such as ALDH activity (Castagnoli et al., 2019). Moreover, modulation of Wnt activity with inhibitors or agonists leads to downregulation and upregulation of PD-L1, respectively (Castagnoli et al., 2019). Notably, despite having achieved promising therapeutic efficacy in many solid tumors, the activity of PD-L1 inhibitors in TNBC remains severely limited (Castagnoli et al., 2019).

## Wnt Signaling and Breast CSCs

One of the central functions of Wnt/β-catenin in stem cell biology is the regulation and maintenance of self-renewal and pluripotency and the governance of differentiation fate. Wnt signaling regulates both maintenance of pluripotency and lineage specification in embryonic stem cells and early embryonic development (de Jaime-Soguero et al., 2018). Small molecule activators of this pathway (GSK3 inhibitors like CHIR99021) are commonly used in embryonic stem cell maintenance culture media (Reya and Clevers, 2005; Nusse, 2008; Zhan et al., 2017; de Jaime-Soguero et al., 2018; Aulicino et al., 2020). Moreover, while it is commonly regarded as a pro-proliferative signaling cascade in several cancer models, its role in proliferation is not linear but rather context-dependent. Studies in embryonic stem cells demonstrate that high Wnt levels correlate with an elongation of cell cycle duration (De Jaime-Soguero et al., 2017).

The involvement of Wnt signaling in the maintenance and regulation of the CSC phenotype has been intensely studied in recent years. Consequently, like in healthy stem cells and embryonic stem cells, it has been shown to regulate a myriad of functions that ultimately contribute to the survival of CSCs, which contributes to drug resistance and relapse.

Both canonical and non-canonical Wnt signaling have been shown to play a central role in promoting breast cancer stemness through CD44 in breast CSCs. Both WNT5A and WNT5B were reported to confer basal-like breast CSC properties by activating canonical and non-canonical Wnt signaling (Shi et al., 2014; Jiang et al., 2019). In agreement, enhanced levels of stabilized β-catenin in TNBC cellular models have shown to increase stemness markers such as pluripotency genes and the total population of ALDH^+^ and CD24^low^/CD44^high^ CSCs promoting resistance to carboplatin (Abreu de Oliveira et al., 2021).

Cell Epithelial to Mesenchymal transition (EMT), which confers metastasizing properties to carcinoma cells, has also been shown to regulate the acquisition of CSC properties through Wnt regulation. Specifically, Snail has been shown to induce resistance to taxol treatment in breast cancer cells by mediating upregulation of miR-125b through the Wnt/β-catenin/TCF7L2 axis. Moreover, miR-125b overexpression was shown to expand CSC populations, and depletion of expression of this miRNA led to the reinstatement of taxanes sensitivity (Liu Z. et al., 2013). Recently, the heat shock family member HSPA9 (mortalin) has been demonstrated to promote EMT and CSC maintenance in breast cancer cell lines via activation of Wnt signaling (Wei et al., 2021). Another recent study further supports the link between EMT and Wnt signaling in the maintenance and/or acquisition of CSC properties. Specifically, Xie and colleagues demonstrated that knockdown of XB130, a cytosolic signal transduction protein found upregulated in breast cancers and associated with poor prognosis, disrupts EMT and Wnt signaling, culminating in decreased CSC activity and tumor-initiating capacity in breast cancer cells (Xie et al., 2019). Zhu and colleagues also demonstrated that BAF chromatin remodeling complex subunit BCL11A expression led to increased tumor formation, cell mobility, tumorsphere forming activity, and EMT, coupled with enhanced Wnt signaling in cancer cell lines (Zhu et al., 2019).

The hypoxic tumor microenvironment also regulates the CSC phenotype through Wnt signaling. Specifically, HIF-2a expression stimulated by hypoxia in breast cancer cells significantly induces paclitaxel resistance. Moreover, HIF-2a exogenous overexpression is accompanied by stem cell marker expression and enhanced Wnt activity. Importantly, inhibition of Wnt signaling by DKK1 reverts stemness and paclitaxel resistance (Yan et al., 2018). More recently, Li and colleagues have also demonstrated the involvement of hypoxia-induced long non-coding RNA RBM5-AS1 in mediating proliferation, migration, invasion, EMT, and stemness maintenance in breast cancer cells under the control of RUNX family transcription factor 2 (RUNX2). Specifically, the authors demonstrated that enrichment of RBM5-AS1 enhanced Wnt signaling by repressing β-catenin degradation and reinforcing the β-catenin:TCF7L2 transcriptional complex (Li et al., 2022).

## Opportunities and Challenges in Wnt Cancer Treatment

The impact of Wnt signaling on tumour initiation, progression, and resistance to treatment is becoming evident, making it a potential therapeutic target for Wnt-driven malignancies and those where Wnt is shown to mediate CSC maintenance and/or treatment adaptation, resistance, and recurrence.

**FIGURE 5:**
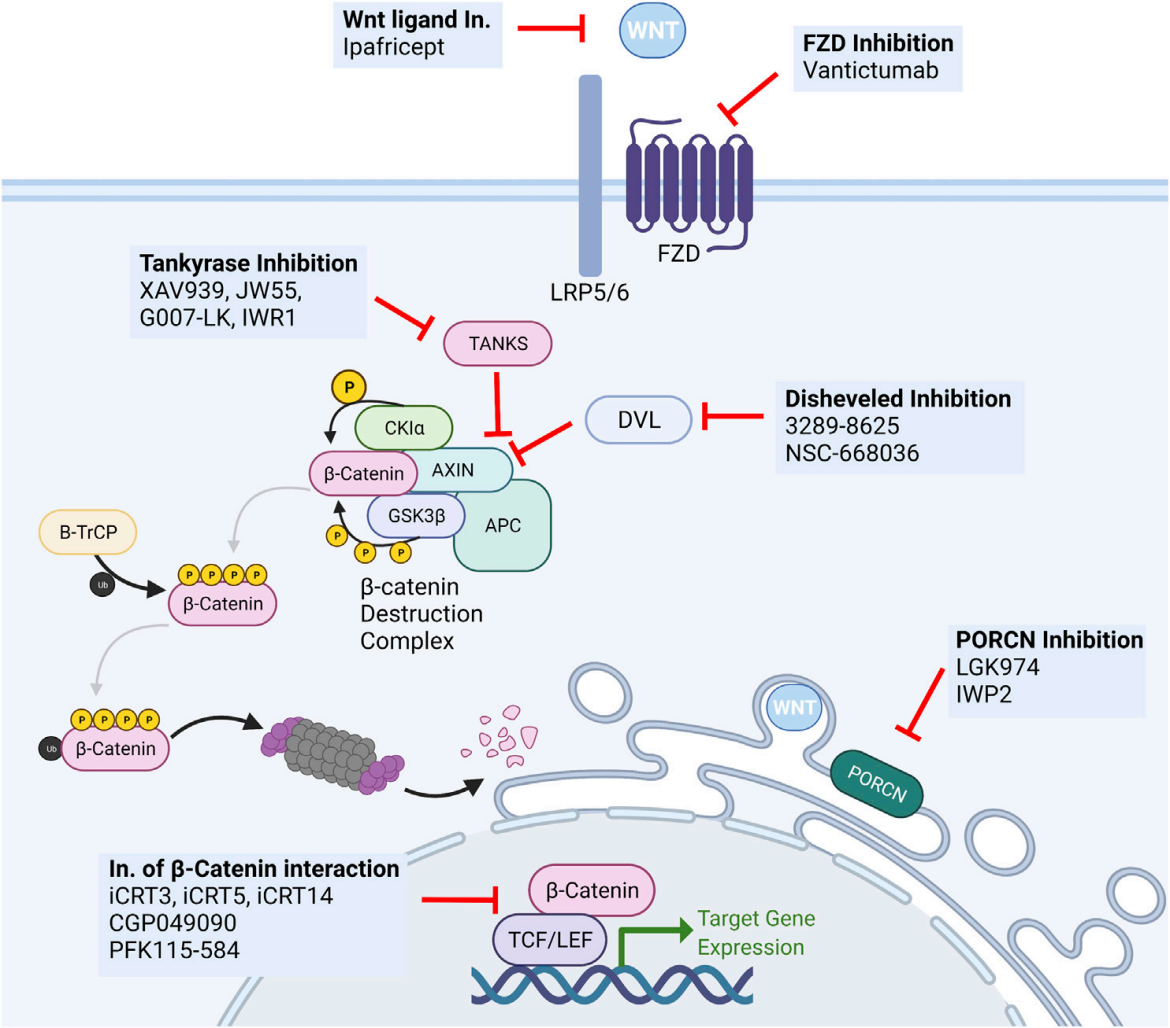
Overview of Wnt targeting drugs. Several classes of biological and small molecule Wnt-targeting drugs have been developed and studied for anti-cancer activity. The most important include monoclonal antibodies targeting secreted Wnt ligands (Ipafricet) or their receptors (Vantictumab), tankyrase inhibitors (XAV939, JW55, G007-LK, IWR1), disheveled inhibitors (3289–8625, NSC-668036), PORCN inhibitors (LGK974, IWP2), and inhibitors of the β-catenin:TCF/LEF transcriptional complex (iCRT3. iCRT5, iCRT14, GGP049090, PFK115-584).

Several pharmacological Wnt inhibitors have been developed to target different members or steps in the signal transduction cascade, from ligand biogenesis to transcriptional activation (Figure 5).

Given the many functions Wnt exerts in normal tissues such as the intestine, hematopoietic system, and skin, the development of efficient and highly specific therapeutic tools is invariably hindered by the challenge of warranting minimal pharmacotoxicity and optimal tolerability. Notably, the intrinsic need of adult tissue stem cells for tight Wnt signaling regulation poses a major hurdle to the clinical feasibility of Wnt signaling inhibition due to the significant hypothetical and demonstrated side effects. One example is the use of tankyrase inhibitors and their severe gastrointestinal side effects (Chatterjee et al., 2021). The same applies to agents targeting other critical developmental signaling pathways such as Notch, Hedgehog, or BMP (Kahn, 2014). As such, despite the intense interest in their study and development, no Wnt-targeting treatments have been approved for human use.

Inhibitors of PORCN have been given substantial attention and tested in various cancers. Given the sheer size of the Wnt and FZD family of ligands and receptors and the high level of complexity of ligand-receptor interactions, the possibility of a “shotgun” approach that disrupts the activity of all ligands is highly desirable. This approach likely prevents compensatory upregulation of alternative receptors or agonistic ligands and the acquisition of mutations leading to alterations in antibody binding affinity. Notwithstanding, the use of such small molecules is always liable to the development of resistance through upregulation drug efflux pumps.

Nonetheless, Liu and colleagues first reported the effectiveness of PORCN inhibitor LGK974 in MMTV-Wnt1 triggered murine breast cancer. Notably, their study reported no relevant intestinal toxicity at the therapeutic dose, with significant tumor reduction (Liu J. et al., 2013). This molecule is currently in phase I/II clinical trials in several solid malignancies, including TNBC (Ghosh et al., 2019; Novartis, 2021). Interestingly, inhibition of PORCN by LGK974 has been shown to disable breast cancer stem cell activity in TNBC and to significantly reverse the acquired carboplatin resistance in the Patient-Derived Xenograft models (Abreu de Oliveira et al., 2021).

In addition to inhibiting their secretion, Wnt ligand activity can be disrupted by Wnt-targeted monoclonal antibodies (ipafricept—OMP54F28) and FZD-targeting monoclonal antibodies (vanctitumab—OMP18R5) (Jimeno et al., 2017; Martin-Orozco et al., 2019; Diamond et al., 2020). Ipafricept (OMP-54F28) is a first-in-class recombinant fusion protein consisting of the Fc domain of IgG1 fused to the Wnt-binding domain of the human FZD8 protein, working as a decoy receptor for Wnt ligands, thereby preventing them from interacting with natural FZD receptors. This fusion protein has shown an impressive response rate in ovarian cancer and is currently in phase Ib clinical trials (Weekes et al., 2016; Diamond et al., 2020). FZD-targeting monoclonal antibodies have also shown therapeutic potential in different cancer types. Vantictumab (OMP18R5) blocks several FZD receptors and inhibits tumor growth in the lung, pancreas, colon, and breast cancer, also showing synergistic activity with taxane treatment. Recently, an anti-FZD7 mAb was shown to drastically enhance the action of the anti-VEGF mAb Bevacizumab in TNBC by targeting FZD7+ cells, thereby inhibiting Wnt/β-catenin-induced EMT and CSC properties (Xie et al., 2021).

Downstream inhibitors of the Wnt signaling pathway have also been given extensive attention. A variety of molecules have been developed to target different intracellular regulators of Wnt signaling. Tankyrase inhibitors such as XAV939, IWR1, JW55, and G007-LK have shown preclinical efficacy but failed to move into clinical trials due to toxicity in preclinical studies (Ghosh et al., 2019). Several small molecules have been developed to inhibit DVL (3289–8625, NSC-668036), resulting in inhibition of Wnt signal transduction (Zhang and Wang, 2020). The inhibition of β-catenin/TCF/LEF-regulated transcription using inhibitors of the transcriptional complex (iCRT3, iCRT5, iCRT14, CGP049090, PFK115-584) has also been granted a good deal of attention. However simple it may sound on paper, this approach has proved to be not so straightforward (Lyou et al., 2017).

One crucial limitation and challenge towards establishing Wnt signaling-targeting drugs as therapeutic options in general and breast cancer specifically is the necessity to identify and stratify in which patients the risk-benefit relationship is reasonable. To that end, the discovery and validation of robust biomarkers to predict response to Wnt-targeted treatments would prove invaluable. Interestingly, a clinical trial has reported a four-gene signature (FBXW2, CCND2, CTBP2, and WIF1) as a predictor of response to paclitaxel/vantictumab combinatorial treatment in HER2 negative breast cancer (van Schie and van Amerongen, 2020). A testament to the challenges of targeting Wnt is that the only molecule currently in clinical development to target Wnt signaling in breast cancer is LGK974 (GlobalData, 2022).

## Conclusion

From the earliest mammary morphogenic events to drug-resistant breast cancer, our knowledge of the importance of Wnt signaling in the breast keeps increasing. The virtual inexistence of well-characterized Wnt-driving genetic mutation casts a shadow over the functional relevance of aberrations in Wnt signaling regulation in breast cancer. Discovering the provenance of such aberrant levels of Wnt signaling activation could prove invaluable in the ongoing efforts to discover druggable targets for TNBC.

Exploiting the relationship between aberrant Wnt activity and enhanced cancer stem cell function and drug resistance has long been regarded as a potential therapeutic avenue for this breast cancer subtype. However, our current understanding of the underlying pathobiology of aberrant Wnt signaling is shallow since so much is yet to be understood about the regulation of this intricate signaling cascade in healthy conditions. Notwithstanding, understanding whether aberrant Wnt signaling is an intrinsic feature of breast cancer, perhaps harbored by underrepresented cancer cell populations, or a feature of populational evolution and treatment adaptation by non-mutational mechanisms is an important question that needs to be addressed to untap the potential for Wnt signaling targeting therapies in breast cancer. Relevant preclinical research models will be critical towards resolving the underlying Wnt signaling heterogeneity of breast cancers. Murine models of breast cancer differ from human breast neoplasms in many ways, including their immune environment, vascularization, and tumor microenvironment, among others. All of these can contribute to the disconnects often observed in the translation of fundamental research studies into the clinic (Roarty and Echeverria, 2021).

Due to the myriad of Wnt-dependent physiologic functions, pharmacological modulation of this pathway remains particularly challenging. As such, mapping the functional outcomes of Wnt signaling perturbations in breast cancer cell populations to dissect possible points of pharmacological intervention is necessary to circumvent organism-wide side effects of Wnt signaling inhibition.

Understanding which patients are more likely to benefit from Wnt targeted therapies is just as important has gaining deeper knowledge of the underlying biology of the Wnt signaling pathway. The selection of such patients for Wnt targeted therapies requires a serious commitment toward the development of stratification protocols based on robust biomarkers and detection assays.

Wnt signaling-targeting therapies could undoubtedly carve out an important place in the breast cancer therapeutic arsenal. However, given the current pharmacological state-of-the-art and the overall complexity of Wnt signaling activation in normal and disease conditions, significant research is needed to enable Wnt-targeting drugs as suitable therapeutic tools.

## References

[B1] Abreu de Oliveira W. A. Moens S. el Laithy Y. van der Veer B. K. Athanasouli P. Cortesi E. E. (2021). Wnt/β-Catenin Inhibition Disrupts Carboplatin Resistance in Isogenic Models of Triple-Negative Breast Cancer. Front. Oncol. 11, 705384. 10.3389/fonc.2021.705384 34367990 PMC8340846

[B2] Aulicino F. Pedone E. Sottile F. Lluis F. Marucci L. Cosma M. P. (2020). Canonical Wnt Pathway Controls mESC Self-Renewal through Inhibition of Spontaneous Differentiation via β-Catenin/TCF/LEF Functions. Stem Cell Rep. 15, 646–661. 10.1016/j.stemcr.2020.07.019 PMC7486219 32822589

[B3] Axelrod J. D. Matsuno K. Artavanis-Tsakonas S. Perrimon N. (1996). Interaction between Wingless and Notch Signaling Pathways Mediated by Dishevelled. Science 271, 1826–1832. 10.1126/science.271.5257.1826 8596950

[B4] Ayyanan A. Civenni G. Ciarloni L. Morel C. Mueller N. Lefort K. (2006). Increased Wnt Signaling Triggers Oncogenic Conversion of Human Breast Epithelial Cells by a Notch-dependent Mechanism. Proc. Natl. Acad. Sci. U.S.A. 103, 3799–3804. 10.1073/pnas.0600065103 16501043 PMC1450156

[B5] Badders N. M. Goel S. Clark R. J. Klos K. S. Kim S. Bafico A. (2009). The Wnt Receptor, Lrp5, Is Expressed by Mouse Mammary Stem Cells and Is Required to Maintain the Basal Lineage. PLoS ONE 4, e6594. 10.1371/journal.pone.0006594 19672307 PMC2720450

[B6] Bergin A. R. T. Loi S. (2019). Triple-negative Breast Cancer: Recent Treatment Advances. F1000Res 8, 1342. 10.12688/f1000research.18888.1 PMC6681627 31448088

[B8] Björklund P. Svedlund J. Olsson A.-K. Åkerström G. Westin G. (2009). The Internally Truncated LRP5 Receptor Presents a Therapeutic Target in Breast Cancer. PLoS ONE 4, e4243. 10.1371/journal.pone.0004243 19158955 PMC2627768

[B10] Boras-Granic K. Chang H. Grosschedl R. Hamel P. A. (2006). Lef1 Is Required for the Transition of Wnt Signaling from Mesenchymal to Epithelial Cells in the Mouse Embryonic Mammary Gland. Dev. Biol. 295, 219–231. 10.1016/j.ydbio.2006.03.030 16678815

[B11] Boras-Granic K. Wysolmerski J. J. (2008). Wnt Signaling in Breast Organogenesis. Organogenesis 4, 116–122. 10.4161/org.4.2.5858 19279723 PMC2634257

[B12] Borri F. Granaglia A. (2021). Pathology of Triple Negative Breast Cancer. Seminars Cancer Biol. 72, 136–145. 10.1016/j.semcancer.2020.06.005 32544511

[B13] Buechling T. Chaudhary V. Spirohn K. Weiss M. Boutros M. (2011). P24 Proteins Are Required for Secretion of Wnt Ligands. EMBO Rep. 12, 1265–1272. 10.1038/embor.2011.212 22094269 PMC3245698

[B14] Bühler T. A. Dale T. C. Kieback C. Humphreys R. C. Rosen J. M. (1993). Localization and Quantification of Wnt-2 Gene Expression in Mouse Mammary Development. Dev. Biol. 155, 87–96. 10.1006/dbio.1993.1009 8416847

[B15] Burgess A. W. (2008). EGFR Family: Structure Physiology Signalling and Therapeutic Targets†. Growth factors. 26, 263–274. 10.1080/08977190802312844 18800267

[B16] Cancer Genome Atlas Network (2012). Comprehensive Molecular Portraits of Human Breast Tumours. Nature 490, 61–70. 10.1038/nature11412 23000897 PMC3465532

[B17] Castagnoli L. Cancila V. Cordoba-Romero S. L. Faraci S. Talarico G. Belmonte B. (2019). WNT Signaling Modulates PD-L1 Expression in the Stem Cell Compartment of Triple-Negative Breast Cancer. Oncogene 38, 4047–4060. 10.1038/s41388-019-0700-2 30705400 PMC6755989

[B18] Chatterjee A. Paul S. Bisht B. Bhattacharya S. Sivasubramaniam S. Paul M. K. (2022). Advances in Targeting the WNT/β-catenin Signaling Pathway in Cancer. Drug Discov. Today 27, 82–101. 10.1016/j.drudis.2021.07.007 34252612

[B19] Chen W. Morabito S. J. Kessenbrock K. Enver T. Meyer K. B. Teschendorff A. E. (2019). Single-cell Landscape in Mammary Epithelium Reveals Bipotent-like Cells Associated with Breast Cancer Risk and Outcome. Commun. Biol. 2, 306. 10.1038/s42003-019-0554-8 31428694 PMC6689007

[B20] Choy L. Hagenbeek T. J. Solon M. French D. Finkle D. Shelton A. (2017). Constitutive NOTCH3 Signaling Promotes the Growth of Basal Breast Cancers. Cancer Res. 77, 1439–1452. 10.1158/0008-5472.CAN-16-1022 28108512

[B21] Chu E. Y. Hens J. Andl T. Kairo A. Yamaguchi T. P. Brisken C. (2004). Canonical WNT Signaling Promotes Mammary Placode Development and Is Essential for Initiation of Mammary Gland Morphogenesis. Development 131, 4819–4829. 10.1242/dev.01347 15342465

[B22] Cicatiello L. Addeo R. Sasso A. Altucci L. Petrizzi V. B. Borgo R. (2004). Estrogens and Progesterone Promote Persistent CCND1 Gene Activation during G 1 by Inducing Transcriptional Derepression via C- Jun /c- Fos /Estrogen Receptor (Progesterone Receptor) Complex Assembly to a Distal Regulatory Element and Recruitment of Cyclin D1 to its Own Gene Promoter. Mol. Cell Biol. 24, 7260–7274. 10.1128/mcb.24.16.7260-7274.2004 15282324 PMC479712

[B23] Colacino J. A. Azizi E. Brooks M. D. Harouaka R. Fouladdel S. McDermott S. P. (2018). Heterogeneity of Human Breast Stem and Progenitor Cells as Revealed by Transcriptional Profiling. Stem Cell Rep. 10, 1596–1609. 10.1016/j.stemcr.2018.03.001 PMC5995162 29606612

[B24] Collu G. M. Brennan K. (2007). Cooperation between Wnt and Notch Signalling in Human Breast Cancer. Breast Cancer Res. 9, 105. 10.1186/bcr1671 17531087 PMC1929088

[B25] Collu G. M. Hidalgo-Sastre A. Brennan K. (2014). Wnt-Notch Signalling Crosstalk in Development and Disease. Cell. Mol. Life Sci. 71, 3553–3567. 10.1007/s00018-014-1644-x 24942883 PMC11113451

[B26] Coudreuse D. Korswagen H. C. (2007). The Making of Wnt: New Insights into Wnt Maturation, Sorting and Secretion. Development 134, 3–12. 10.1242/dev.02699 17138665

[B27] Cruciat C.-M. Niehrs C. (2013). Secreted and Transmembrane Wnt Inhibitors and Activators. Cold Spring Harb. Perspect. Biol. 5, a015081. 10.1101/cshperspect.a015081 23085770 PMC3578365

[B28] Curtis C. Shah S. P. Shah S. P. Chin S.-F. Turashvili G. Rueda O. M. (2012). The Genomic and Transcriptomic Architecture of 2,000 Breast Tumours Reveals Novel Subgroups. Nature 486, 346–352. 10.1038/nature10983 22522925 PMC3440846

[B29] Dai X. Li T. Bai Z. Yang Y. Liu X. Zhan J. (2015). Breast Cancer Intrinsic Subtype Classification, Clinical Use and Future Trends. Am. J. Cancer Res. 5, 2929–2943. 10.1534/g3.114.014894 26693050 PMC4656721

[B30] de Jaime-Soguero A. Abreu de Oliveira W. Lluis F. (2018). The Pleiotropic Effects of the Canonical Wnt Pathway in Early Development and Pluripotency. Genes 9, 93–23. 10.3390/genes9020093 PMC5852589 29443926

[B31] De Jaime-Soguero A. Aulicino F. Ertaylan G. Griego A. Cerrato A. Tallam A. (2017). Wnt/Tcf1 Pathway Restricts Embryonic Stem Cell Cycle through Activation of the Ink4/Arf Locus. PLoS Genet. 13, e1006682. 10.1371/journal.pgen.1006682 28346462 PMC5386305

[B32] de Lau W. Peng W. C. Gros P. Clevers H. (2014). The R-spondin/Lgr5/Rnf43 Module: Regulator of Wnt Signal Strength. Genes Dev. 28, 305–316. 10.1101/gad.235473.113 24532711 PMC3937510

[B33] Denkert C. Liedtke C. Tutt A. von Minckwitz G. (2017). Molecular Alterations in Triple-Negative Breast Cancer-The Road to New Treatment Strategies. Lancet 389, 2430–2442. 10.1016/S0140-6736(16)32454-0 27939063

[B34] Dent R. Trudeau M. Pritchard K. I. Hanna W. M. Kahn H. K. Sawka C. A. (2007). Triple-Negative Breast Cancer: Clinical Features and Patterns of Recurrence. Clin. Cancer Res. 13, 4429–4434. 10.1158/1078-0432.CCR-06-3045 17671126

[B35] Dey N. Barwick B. G. Moreno C. S. Ordanic-Kodani M. Chen Z. Oprea-Ilies G. (2013). Wnt Signaling in Triple Negative Breast Cancer Is Associated with Metastasis. BMC Cancer 13, 537. 10.1186/1471-2407-13-537 24209998 PMC4226307

[B36] Diamond J. R. Becerra C. Richards D. Mita A. Osborne C. O’Shaughnessy J. (2020). Phase Ib Clinical Trial of the Anti-frizzled Antibody Vantictumab (OMP-18R5) Plus Paclitaxel in Patients with Locally Advanced or Metastatic HER2-Negative Breast Cancer. Breast Cancer Res. Treat. 184, 53–62. 10.1007/s10549-020-05817-w 32803633 PMC7572714

[B37] Dontu G. Ince T. A. (2015). Of Mice and Women: A Comparative Tissue Biology Perspective of Breast Stem Cells and Differentiation. J. Mammary Gland. Biol. Neoplasia 20, 51–62. 10.1007/s10911-015-9341-4 26286174 PMC4595531

[B38] Early Breast Cancer Trialists’ Collaborative Group (EBCTCG) (2005). Effects of Chemotherapy and Hormonal Therapy for Early Breast Cancer on Recurrence and 15-year Survival: an Overview of the Randomised Trials. Lancet 365, 1687–1717. 10.1016/S0140-6736(05)66544-0 15894097

[B39] Echeverria G. V. Ge Z. Seth S. Zhang X. Jeter-Jones S. Zhou X. (2019). Resistance to Neoadjuvant Chemotherapy in Triple-Negative Breast Cancer Mediated by a Reversible Drug-Tolerant State. Sci. Transl. Med. 11, eaav0936. 10.1126/scitranslmed.aav0936 30996079 PMC6541393

[B40] Emons G. Spitzner M. Reineke S. Möller J. Auslander N. Kramer F. (2017). Chemoradiotherapy Resistance in Colorectal Cancer Cells Is Mediated by Wnt/β-Catenin Signaling. Mol. Cancer Res. 15, 1481–1490. 10.1158/1541-7786.MCR-17-0205 28811361 PMC5772978

[B41] Feng Y. Spezia M. Huang S. Yuan C. Zeng Z. Zhang L. (2018). Breast Cancer Development and Progression: Risk Factors, Cancer Stem Cells, Signaling Pathways, Genomics, and Molecular Pathogenesis. Genes & Dis. 5, 77–106. 10.1016/j.gendis.2018.05.001 PMC6147049 30258937

[B42] Foley J. Dann P. Hong J. Cosgrove J. Dreyer B. Rimm D. (2001). Parathyroid Hormone-Related Protein Maintains Mammary Epithelial Fate and Triggers Nipple Skin Differentiation during Embryonic Breast Development. Development 128, 513–525. 10.1242/dev.128.4.513 11171335

[B43] Foulkes W. D. Smith I. E. Reis-Filho J. S. (2010). Triple-Negative Breast Cancer. N. Engl. J. Med. 363, 1938–1948. 10.1056/NEJMra1001389 21067385

[B44] Fukumoto T. Zhu H. Nacarelli T. Karakashev S. Fatkhutdinov N. Wu S. (2019). N6-methylation of Adenosine (m6A) of FZD10 mRNA Contributes to PARP Inhibitor Resistance. Cancer Res. 79, 2812–2820. 10.1158/0008-5472.CAN-18-3592 30967398 PMC6548690

[B45] Gao Y. Liu Z. Zhang X. He J. Pan Y. Hao F. (2013). Inhibition of Cytoplasmic GSK-3β Increases Cisplatin Resistance through Activation of Wnt/β-Catenin Signaling in A549/DDP Cells. Cancer Lett. 336, 231–239. 10.1016/j.canlet.2013.05.005 23673211

[B46] Geyer F. C. Lacroix-Triki M. Savage K. Arnedos M. Lambros M. B. MacKay A. (2011). β-Catenin Pathway Activation in Breast Cancer Is Associated with Triple-Negative Phenotype but Not with CTNNB1 Mutation. Mod. Pathol. 24, 209–231. 10.1038/modpathol.2010.205 21076461

[B47] Ghosh N. Hossain U. Mandal A. Sil P. C. (2019). The Wnt Signaling Pathway: a Potential Therapeutic Target against Cancer. Ann. N.Y. Acad. Sci. 1443, 54–74. 10.1111/nyas.14027 31017675

[B48] Ginestier C. Hur M. H. Charafe-Jauffret E. Monville F. Dutcher J. Brown M. (2007). ALDH1 Is a Marker of Normal and Malignant Human Mammary Stem Cells and a Predictor of Poor Clinical Outcome. Cell Stem Cell 1, 555–567. 10.1016/j.stem.2007.08.014 18371393 PMC2423808

[B49] GlobalData (2022). Clinical Trials Database.

[B50] Guan J. Zhou W. Hafner M. Blake R. A. Chalouni C. Chen I. P. (2019). Therapeutic Ligands Antagonize Estrogen Receptor Function by Impairing its Mobility. Cell 178, 949–963. e18. 10.1016/j.cell.2019.06.026 31353221

[B51] Hallett R. M. Kondratyev M. K. Giacomelli A. O. Nixon A. M. L. Girgis-Gabardo A. Ilieva D. (2012). Small Molecule Antagonists of the Wnt/Beta-Catenin Signaling Pathway Target Breast Tumor-Initiating Cells in a Her2/Neu Mouse Model of Breast Cancer. PLoS One 7, e33976. 10.1371/JOURNAL.PONE.0033976 22470504 PMC3314694

[B52] Harbeck N. Penault-Llorca F. Cortes J. Gnant M. Houssami N. Poortmans P. (2019). Breast Cancer. Nat. Rev. Dis. Prim. 5, 66. 10.1038/s41572-019-0111-2 31548545

[B53] Hashizume R. Koizumi H. Ihara A. Ohta T. Uchikoshi T. (1996). Expression of β-catenin in Normal Breast Tissue and Breast Carcinoma: a Comparative Study with Epithelial Cadherin and α-catenin. Histopathology 29, 139–146. 10.1046/j.1365-2559.1996.d01-499.x 8872147

[B54] Hayward P. Balayo T. Martinez Arias A. (2006). Notch Synergizes with Axin to Regulate the Activity of Armadillo in Drosophila. Dev. Dyn. 235, 2656–2666. 10.1002/dvdy.20902 16881048

[B55] Hiremath M. Dann P. Fischer J. Butterworth D. Boras-Granic K. Hens J. (2012). Parathyroid Hormone-Related Protein Activates Wnt Signaling to Specify the Embryonic Mammary Mesenchyme. Dev. Camb. 139, 4239–4249. 10.1242/dev.080671 PMC3478689 23034629

[B56] Hou M.-F. Chen P.-M. Chu P.-Y. (2018). LGR5 Overexpression Confers Poor Relapse-free Survival in Breast Cancer Patients. BMC Cancer 18, 1–8. 10.1186/s12885-018-4018-1 29471794 PMC5824537

[B57] Howe L. R. Brown A. M. C. (2004). Wnt Signaling and Breast Cancer. Cancer Biol. Ther. 3, 36–41. 10.4161/cbt.3.1.561 14739782

[B58] Hu C. Diévart A. Lupien M. Calvo E. Tremblay G. Jolicoeur P. (2006). Overexpression of Activated Murine Notch1 and Notch3 in Transgenic Mice Blocks Mammary Gland Development and Induces Mammary Tumors. Am. J. Pathology 168, 973–990. 10.2353/ajpath.2006.050416 PMC1606519 16507912

[B59] Hubalek M. Czech T. Müller H. (2017). Biological Subtypes of Triple-Negative Breast Cancer. Breast Care 12, 8–14. 10.1159/000455820 28611535 PMC5465739

[B60] Huguet E. L. McMahon J. A. McMahon A. P. Bicknell R. Harris A. L. (1994). Differential Expression of Human Wnt Genes 2, 3, 4, and 7B in Human Breast Cell Lines and Normal and Disease States of Human Breast Tissue. Cancer Res. 54, 2615–2621. 8168088

[B61] Imatani A. Callahan R. (2000). Identification of a Novel NOTCH-4/INT-3 RNA Species Encoding an Activated Gene Product in Certain Human Tumor Cell Lines. Oncogene 19, 223–231. 10.1038/sj.onc.1203295 10645000

[B62] Inman J. L. Robertson C. Mott J. D. Bissell M. J. (2015). Mammary Gland Development: Cell Fate Specification, Stem Cells and the Microenvironment. Dev. Camb. 142, 1028–1042. 10.1242/dev.087643 25758218

[B63] Itasaki N. Hoppler S. (2009). Crosstalk between Wnt and Bone Morphogenic Protein Signaling: A Turbulent Relationship. Dev. Dyn. 239, NA. 10.1002/dvdy.22009 19544585

[B64] Ithimakin S. Day K. C. Malik F. Zen Q. Dawsey S. J. Bersano-Begey T. F. (2013). HER2 Drives Luminal Breast Cancer Stem Cells in the Absence of HER2 Amplification: Implications for Efficacy of Adjuvant Trastuzumab. Cancer Res. 73, 1635–1646. 10.1158/0008-5472.CAN-12-3349 23442322 PMC3600586

[B65] Jiang S. Zhang M. Zhang Y. Zhou W. Zhu T. Ruan Q. (2019). WNT5B Governs the Phenotype of Basal-like Breast Cancer by Activating WNT Signaling. Cell Commun. Signal 17, 1–19. 10.1186/s12964-019-0419-2 31462314 PMC6714433

[B66] Jimeno A. Gordon M. Chugh R. Messersmith W. Mendelson D. Dupont J. (2017). A First-In-Human Phase I Study of the Anticancer Stem Cell Agent Ipafricept (OMP-54F28), a Decoy Receptor for Wnt Ligands, in Patients with Advanced Solid Tumors. Clin. Cancer Res. 23, 7490–7497. 10.1158/1078-0432.CCR-17-2157 28954784

[B67] Kahn M. (2014). Can We Safely Target the WNT Pathway? Nat. Rev. Drug Discov. 13, 513–532. 10.1038/nrd4233 24981364 PMC4426976

[B68] Kakugawa S. Langton P. F. Zebisch M. Howell S. A. Chang T.-H. Liu Y. (2015). Notum Deacylates Wnt Proteins to Suppress Signalling Activity. Nature 519 (7542), 187–192. 10.1038/nature14259 25731175 PMC4376489

[B69] Kanwar S. S. Yu Y. Nautiyal J. Patel B. B. Majumdar A. P. (2010). The Wnt/β-Catenin Pathway Regulates Growth and Maintenance of Colonospheres. Mol. Cancer 9, 212. 10.1186/1476-4598-9-212 20691072 PMC2924313

[B70] Khramtsov A. I. Khramtsova G. F. Tretiakova M. Huo D. Olopade O. I. Goss K. H. (2010). Wnt/β-Catenin Pathway Activation Is Enriched in Basal-like Breast Cancers and Predicts Poor Outcome. Am. J. Pathology 176, 2911–2920. 10.2353/ajpath.2010.091125 PMC2877852 20395444

[B71] Kim C. Gao R. Sei E. Brandt R. Hartman J. Hatschek T. (2018). Chemoresistance Evolution in Triple-Negative Breast Cancer Delineated by Single-Cell Sequencing. Cell 173, 879–893. e13. 10.1016/J.CELL.2018.03.041 29681456 PMC6132060

[B72] Kinzler K. W. Nilbert M. C. Su L.-K. Vogelstein B. Bryan T. M. Levy D. B. (1991). Identification of FAP Locus Genes from Chromosome 5q21. Science 253, 661–665. 10.1126/science.1651562 1651562

[B73] Korinek V. Barker N. Morin P. J. Van Wichen D. De Weger R. Kinzler K. W. (1997). Constitutive Transcriptional Activation by a β-Catenin-Tcf Complex in APC −/− Colon Carcinoma. Science 275, 1784–1787. 10.1126/science.275.5307.1784 9065401

[B74] Korkaya H. Paulson A. Charafe-Jauffret E. Ginestier C. Brown M. Dutcher J. (2009). Regulation of Mammary Stem/Progenitor Cells by PTEN/Akt/β-Catenin Signaling. PLoS Biol. 7, e1000121. 10.1371/JOURNAL.PBIO.1000121 19492080 PMC2683567

[B75] Korkaya H. Paulson A. Iovino F. Wicha M. S. (2008). HER2 Regulates the Mammary Stem/progenitor Cell Population Driving Tumorigenesis and Invasion. Oncogene 27, 6120–6130. 10.1038/onc.2008.207 18591932 PMC2602947

[B76] Kouros-Mehr H. Werb Z. (2006). Candidate Regulators of Mammary Branching Morphogenesis Identified by Genome-wide Transcript Analysis. Dev. Dyn. 235, 3404–3412. 10.1002/dvdy.20978 17039550 PMC2730892

[B77] Koval A. Katanaev V. L. (2018). Dramatic Dysbalancing of the Wnt Pathway in Breast Cancers. Sci. Rep. 8, 2–11. 10.1038/s41598-018-25672-6 29743726 PMC5943245

[B78] Kovall R. A. Gebelein B. Sprinzak D. Kopan R. (2017). The Canonical Notch Signaling Pathway: Structural and Biochemical Insights into Shape, Sugar, and Force. Dev. Cell 41, 228–241. 10.1016/j.devcel.2017.04.001 28486129 PMC5492985

[B79] Lee C. W. Simin K. Liu Q. Plescia J. Guha M. Khan A. (2008). A Functional Notch-Survivin Gene Signature in Basal Breast Cancer. Breast Cancer Res. 10, R97. 10.1186/bcr2200 19025652 PMC2656893

[B80] Lee K. J. Mann E. Wright G. Piett C. G. Nagel Z. D. Gassman N. R. (2020). Exploiting DNA Repair Defects in Triple Negative Breast Cancer to Improve Cell Killing. Ther. Adv. Med. Oncol. 12, 175883592095835. 10.1177/1758835920958354 PMC7502856 32994807

[B82] Lehmann B. D. Bauer J. A. Chen X. Sanders M. E. Chakravarthy A. B. Shyr Y. (2011). Identification of Human Triple-Negative Breast Cancer Subtypes and Preclinical Models for Selection of Targeted Therapies. J. Clin. Invest. 121, 2750–2767. 10.1172/JCI45014 21633166 PMC3127435

[B83] Lehmann B. D. Jovanović B. Chen X. Estrada M. v. Johnson K. N. Shyr Y. (2016). Refinement of Triple-Negative Breast Cancer Molecular Subtypes: Implications for Neoadjuvant Chemotherapy Selection. PLoS ONE 11, e0157368–22. 10.1371/journal.pone.0157368 27310713 PMC4911051

[B84] Li C. Heidt D. G. Dalerba P. Burant C. F. Zhang L. Adsay V. (2007). Identification of Pancreatic Cancer Stem Cells. Cancer Res. 67, 1030–1037. 10.1158/0008-5472.CAN-06-2030 17283135

[B85] Li X. Xiang Y. Li F. Yin C. Li B. Ke X. (2019). WNT/β-Catenin Signaling Pathway Regulating T Cell-Inflammation in the Tumor Microenvironment. Front. Immunol. 10, 1–12. 10.3389/fimmu.2019.02293 31616443 PMC6775198

[B86] Li X. Yang J. Ni R. Chen J. Zhou Y. Song H. (2022). Hypoxia-induced lncRNA RBM5-AS1 Promotes Tumorigenesis via Activating Wnt/β-Catenin Signaling in Breast Cancer. Cell Death Dis. 13, 95. 10.1038/s41419-022-04536-y 35110544 PMC8810931

[B87] Liu J. Pan S. Hsieh M. H. Ng N. Sun F. Wang T. (2013a). Targeting Wnt-Driven Cancer through the Inhibition of Porcupine by LGK974. Proc. Natl. Acad. Sci. U.S.A. 110, 20224–20229. 10.1073/pnas.1314239110 24277854 PMC3864356

[B88] Liu Z. Liu H. Desai S. Schmitt D. C. Zhou M. Khong H. T. (2013b). MiR-125b Functions as a Key Mediator for Snail-Induced Stem Cell Propagation and Chemoresistance. J. Biol. Chem. 288, 4334–4345. 10.1074/jbc.M112.419168 23255607 PMC3567684

[B89] Loh Y. N. Hedditch E. L. Baker L. A. Jary E. Ward R. L. Ford C. E. (2013). The Wnt Signalling Pathway Is Upregulated in an *In Vitro* Model of Acquired Tamoxifen Resistant Breast Cancer. BMC Cancer 13, 174. 10.1186/1471-2407-13-174 23547709 PMC3621642

[B90] Loi S. Criscitiello C. Fumagalli D. Saini K. S. (2010). Tamoxifen in Early-Stage Estrogen Receptor-Positive Breast Cancer: Overview of Clinical Use and Molecular Biomarkers for Patient Selection. Ott 1, 1. 10.2147/OTT.S10155 PMC3084302 21552410

[B92] Loibl S. Poortmans P. Morrow M. Denkert C. Curigliano G. (2021). Breast Cancer. Lancet 397, 1750–1769. 10.1016/S0140-6736(20)32381-3 33812473

[B93] Luga V. Zhang L. Viloria-Petit A. M. Ogunjimi A. A. Inanlou M. R. Chiu E. (2012). Exosomes Mediate Stromal Mobilization of Autocrine Wnt-PCP Signaling in Breast Cancer Cell Migration. Cell 151, 1542–1556. 10.1016/j.cell.2012.11.024 23260141

[B94] Lyou Y. Habowski A. N. Chen G. T. Waterman M. L. (2017). Inhibition of Nuclear Wnt Signalling: Challenges of an Elusive Target for Cancer Therapy. Br. J. Pharmacol. 174, 4589–4599. 10.1111/bph.13963 28752891 PMC5727325

[B95] Ma J. Lu W. Chen D. Xu B. Li Y. (2017). Role of Wnt Co-receptor LRP6 in Triple Negative Breast Cancer Cell Migration and Invasion. J. Cell. Biochem. 118, 2968–2976. 10.1002/jcb.25956 28247948 PMC10928515

[B97] Manzo G. (2019). Similarities between Embryo Development and Cancer Process Suggest New Strategies for Research and Therapy of Tumors: A New Point of View. Front. Cell Dev. Biol. 7, 1–18. 10.3389/fcell.2019.00020 30899759 PMC6416183

[B98] Marra A. Trapani D. Viale G. Criscitiello C. Curigliano G. (2020). Practical Classification of Triple-Negative Breast Cancer: Intratumoral Heterogeneity, Mechanisms of Drug Resistance, and Novel Therapies. npj Breast Cancer 6, 1–16. 10.1038/s41523-020-00197-2 33088912 PMC7568552

[B99] Martin-Orozco E. Sanchez-Fernandez A. Ortiz-Parra I. Ayala-San Nicolas M. (2019). WNT Signaling in Tumors: The Way to Evade Drugs and Immunity. Front. Immunol. 10, 1–21. 10.3389/fimmu.2019.02854 31921125 PMC6934036

[B100] Masuda H. Baggerly K. A. Wang Y. Zhang Y. Gonzalez-Angulo A. M. Meric-Bernstam F. (2013). Differential Response to Neoadjuvant Chemotherapy Among 7 Triple-Negative Breast Cancer Molecular Subtypes. Clin. Cancer Res. 19, 5533–5540. 10.1158/1078-0432.CCR-13-0799 23948975 PMC3813597

[B101] Medina D. (1996). The Mammary Gland: a Unique Organ for the Study of Development and Tumorigenesis. J. Mammary Gland. Biol. Neoplasia 1, 5–19. 10.1007/BF02096299 10887477

[B102] Meyers S. L. O'Brien M. T. Smith T. Dudley J. P. (1990). Analysis of the Int-1, Int-2, C-Myc, and Neu Oncogenes in Human Breast Carcinomas. Cancer Res. 50, 5911–5918. 1975511

[B103] Mikels A. J. Nusse R. (2006). Wnts as Ligands: Processing, Secretion and Reception. Oncogene 25, 7461–7468. 10.1038/sj.onc.1210053 17143290

[B106] Morin P. J. Sparks A. B. Korinek V. Barker N. Clevers H. Vogelstein B. (1997). Activation of β-Catenin-Tcf Signaling in Colon Cancer by Mutations in β-Catenin or APC. Science 275, 1787–1790. 10.1126/science.275.5307.1787 9065402

[B107] Morris S.-A. L. Huang S. (2016). Crosstalk of the Wnt/β-Catenin Pathway with Other Pathways in Cancer Cells. Genes & Dis. 3, 41–47. 10.1016/j.gendis.2015.12.003 PMC4828918 27081668

[B108] Muñoz Descalzo S. Martinez Arias A. (2012). The Structure of Wntch Signalling and the Resolution of Transition States in Development. Seminars Cell & Dev. Biol. 23, 443–449. 10.1016/j.semcdb.2012.01.012 22326376

[B109] Muñoz-Descalzo S. Sanders P. G. T. Montagne C. Johnson R. I. Balayo T. Arias A. M. (2010). Wingless Modulates the Ligand Independent Traffic of Notch through Dishevelled. Fly 4, 182–193. 10.4161/fly.4.3.11998 20495361

[B110] Nagano H. Tomimaru Y. Eguchi H. Hama N. Wada H. Kawamoto K. (2013). MicroRNA-29a Induces Resistance to Gemcitabine through the Wnt/β-Catenin Signaling Pathway in Pancreatic Cancer Cells. Int. J. Oncol. 43, 1066–1072. 10.3892/ijo.2013.2037 23900458

[B111] Nishisho I. Nakamura Y. Miyoshi Y. Miki Y. Ando H. Horii A. (1991). Mutations of Chromosome 5q21 Genes in FAP and Colorectal Cancer Patients. Science 253, 665–669. 10.1126/science.1651563 1651563

[B112] Novartis (2021). A Study of LGK974 in Patients with Malignancies Dependent on Wnt Ligands. ClinicalTrials.gov identifier (NCT01351103). Available at: https://clinicaltrials.gov/ct2/show/NCT01351103?id=NCT01351103&draw=2&rank=1&load=cart.

[B113] Nusse R. Clevers H. (2017). Wnt/β-Catenin Signaling, Disease, and Emerging Therapeutic Modalities. Cell 169, 985–999. 10.1016/j.cell.2017.05.016 28575679

[B114] Nusse R. Van Ooyen A. Cox D. Fung Y. K. T. Varmus H. (1984). Mode of Proviral Activation of a Putative Mammary Oncogene (Int-1) on Mouse Chromosome 15. Nature 307, 131–136. 10.1038/307131a0 6318122

[B115] Nusse R. (2008). Wnt Signaling and Stem Cell Control. Cell Res. 18, 523–527. 10.1038/cr.2008.47 18392048

[B116] Nüsslein-Volhard C. Wieschaus E. (1980). Mutations Affecting Segment Number and Polarity in Drosophila. Nature 287, 795–801. 10.1038/287795a0 6776413

[B117] Paine I. S. Lewis M. T. (2017). The Terminal End Bud: the Little Engine that Could. J. Mammary Gland. Biol. Neoplasia 22, 93–108. 10.1007/s10911-017-9372-0 28168376 PMC5488158

[B118] Pandya S. Moore R. G. (2011). Breast Development and Anatomy. Clin. Obstetrics Gynecol. 54, 91–95. 10.1097/GRF.0b013e318207ffe9 21278507

[B119] Park J. H. Ahn J.-H. Kim S.-B. (2018). How Shall We Treat Early Triple-Negative Breast Cancer (TNBC): From the Current Standard to Upcoming Immuno-Molecular Strategies. ESMO Open 3, e000357–16. 10.1136/esmoopen-2018-000357 29765774 PMC5950702

[B120] Perou C. M. Sørlie T. Eisen M. B. van de Rijn M. Jeffrey S. S. Rees C. A. (2000). Molecular Portraits of Human Breast Tumours. Nature 406, 747–752. 10.1038/35021093 10963602

[B121] Piva M. Domenici G. Iriondo O. Rábano M. Simões B. M. Comaills V. (2014). Sox2 Promotes Tamoxifen Resistance in Breast Cancer Cells. EMBO Mol. Med. 6, 66–79. 10.1002/emmm.201303411 24178749 PMC3936493

[B122] Pohl S.-G. Brook N. Agostino M. Arfuso F. Kumar A. P. Dharmarajan A. (2017). Wnt Signaling in Triple-Negative Breast Cancer. Oncogenesis 6, e310. 10.1038/oncsis.2017.14 28368389 PMC5520491

[B123] Prasad C. P. Mirza S. Sharma G. Prashad R. DattaGupta S. Rath G. (2008). Epigenetic Alterations of CDH1 and APC Genes: Relationship with Activation of Wnt/β-Catenin Pathway in Invasive Ductal Carcinoma of Breast. Life Sci. 83, 318–325. 10.1016/j.lfs.2008.06.019 18662704

[B124] Ranganathan P. Weaver K. L. Capobianco A. J. (2011). Notch Signalling in Solid Tumours: a Little Bit of Everything but Not All the Time. Nat. Rev. Cancer 11, 338–351. 10.1038/nrc3035 21508972

[B125] Reya T. Clevers H. (2005). Wnt Signalling in Stem Cells and Cancer. Nature 434, 843–850. 10.1038/nature03319 15829953

[B126] Rijsewijk F. Schuermann M. Wagenaar E. Parren P. Weigel D. Nusse R. (1987). The Drosophila Homology of the Mouse Mammary Oncogene Int-1 Is Identical to the Segment Polarity Gene Wingless. Cell 50, 649–657. 10.1016/0092-8674(87)90038-9 3111720

[B127] Rios A. C. Fu N. Y. Lindeman G. J. Visvader J. E. (2014). *In Situ* identification of Bipotent Stem Cells in the Mammary Gland. Nature 506, 322–327. 10.1038/nature12948 24463516

[B128] Roarty K. Echeverria G. v. (2021). Laboratory Models for Investigating Breast Cancer Therapy Resistance and Metastasis. Front. Oncol. 11, 645698. 10.3389/fonc.2021.645698 33777805 PMC7988094

[B129] Roarty K. Shore A. N. Creighton C. J. Rosen J. M. (2015). Ror2 Regulates Branching, Differentiation, and Actin-Cytoskeletal Dynamics within the Mammary Epithelium. J. Cell Biol. 208, 351–366. 10.1083/jcb.201408058 25624393 PMC4315251

[B130] Robinson D. R. Kalyana-Sundaram S. Wu Y.-M. Shankar S. Cao X. Ateeq B. (2011). Functionally Recurrent Rearrangements of the MAST Kinase and Notch Gene Families in Breast Cancer. Nat. Med. 17, 1646–1651. 10.1038/nm.2580 22101766 PMC3233654

[B131] Rubinfeld B. Souza B. Albert I. Müller O. Chamberlain S. H. Masiarz F. R. (1993). Association of the APC Gene Product with β-Catenin. Science 262, 1731–1734. 10.1126/science.8259518 8259518

[B133] Ryu W.-J. Lee J. D. Park J.-C. Cha P.-H. Cho Y.-H. Kim J. Y. (2020). Destabilization of β-catenin and RAS by Targeting the Wnt/β-Catenin Pathway as a Potential Treatment for Triple-Negative Breast Cancer. Exp. Mol. Med. 52, 832–842. 10.1038/s12276-020-0440-y 32457491 PMC7272395

[B134] Shah D. Osipo C. (2016). Cancer Stem Cells and HER2 Positive Breast Cancer: The Story So Far. Genes & Dis. 3, 114–123. 10.1016/j.gendis.2016.02.002 PMC6095671 30123819

[B135] Shen M. Kang Y. (2018). Complex Interplay between Tumor Microenvironment and Cancer Therapy. Front. Med. 12, 426–439. 10.1007/s11684-018-0663-7 30097962

[B136] Shi J. Wang Y. Zeng L. Wu Y. Deng J. Zhang Q. (2014). Disrupting the Interaction of BRD4 with Diacetylated Twist Suppresses Tumorigenesis in Basal-like Breast Cancer. Cancer Cell 25, 210–225. 10.1016/j.ccr.2014.01.028 24525235 PMC4004960

[B137] Shimizu T. Kagawa T. Inoue T. Nonaka A. Takada S. Aburatani H. (2008). Stabilized β-Catenin Functions through TCF/LEF Proteins and the Notch/RBP-Jκ Complex to Promote Proliferation and Suppress Differentiation of Neural Precursor Cells. Mol. Cell Biol. 28, 7427–7441. 10.1128/MCB.01962-07 18852283 PMC2593432

[B138] Sørlie T. Perou C. M. Tibshirani R. Aas T. Geisler S. Johnsen H. (2001). Gene Expression Patterns of Breast Carcinomas Distinguish Tumor Subclasses with Clinical Implications. Proc. Natl. Acad. Sci. U.S.A. 98 (19), 10869–10874. 10.1073/pnas.191367098 11553815 PMC58566

[B139] Sotiriou C. Neo S.-Y. McShane L. M. Korn E. L. Long P. M. Jazaeri A. (2003). Breast Cancer Classification and Prognosis Based on Gene Expression Profiles from a Population-Based Study. Proc. Natl. Acad. Sci. U.S.A. 100, 10393–10398. 10.1073/pnas.1732912100 12917485 PMC193572

[B140] Stingl J. Eirew P. Ricketson I. Shackleton M. Vaillant F. Choi D. (2006). Purification and Unique Properties of Mammary Epithelial Stem Cells. Nature 439, 993–997. 10.1038/nature04496 16395311

[B141] Su L.-K. Vogelstein B. Kinzler K. W. (1993). Association of the APC Tumor Suppressor Protein with Catenins. Science 262, 1734–1737. 10.1126/science.8259519 8259519

[B142] Sun Y. Campisi J. Higano C. Beer T. M. Porter P. Coleman I. (2012). Treatment-induced Damage to the Tumor Microenvironment Promotes Prostate Cancer Therapy Resistance through WNT16B. Nat. Med. 18, 1359–1368. 10.1038/nm.2890 22863786 PMC3677971

[B143] Sung H. Ferlay J. Siegel R. L. Laversanne M. Soerjomataram I. Jemal A. (2021). Global Cancer Statistics 2020: GLOBOCAN Estimates of Incidence and Mortality Worldwide for 36 Cancers in 185 Countries. CA A Cancer J. Clin. 71, 209–249. 10.3322/caac.21660 33538338

[B144] Tocci J. M. Felcher C. M. García Solá M. E. Kordon E. C. (2020). R‐spondin‐mediated WNT Signaling Potentiation in Mammary and Breast Cancer Development. IUBMB Life 72, 1546–1559. 10.1002/iub.2278 32233118

[B145] Trejo C. L. Luna G. Dravis C. Spike B. T. Wahl G. M. (2017). Lgr5 Is a Marker for Fetal Mammary Stem Cells, but Is Not Essential for Stem Cell Activity or Tumorigenesis. npj Breast Cancer 3, 1–10. 10.1038/s41523-017-0018-6 28649656 PMC5460261

[B146] Tsukamoto A. S. Grosschedl R. Guzman R. C. Parslow T. Varmus H. E. (1988). Expression of the Int-1 Gene in Transgenic Mice Is Associated with Mammary Gland Hyperplasia and Adenocarcinomas in Male and Female Mice. Cell 55, 619–625. 10.1016/0092-8674(88)90220-6 3180222

[B147] Ugolini F. Charafe-Jauffret E. Bardou V.-J. Geneix J. Adélaïde J. Labat-Moleur F. (2001). WNT Pathway and Mammary Carcinogenesis: Loss of Expression of Candidate Tumor Suppressor Gene SFRP1 in Most Invasive Carcinomas except of the Medullary Type. Oncogene 20, 5810–5817. 10.1038/sj.onc.1204706 11593386

[B148] van Schie E. H. van Amerongen R. (2020). Aberrant WNT/CTNNB1 Signaling as a Therapeutic Target in Human Breast Cancer: Weighing the Evidence. Front. Cell Dev. Biol. 8, 1–14. 10.3389/fcell.2020.00025 32083079 PMC7005411

[B149] Veltmaat J. M. Van Veelen W. Thiery J. P. Bellusci S. (2004). Identification of the Mammary Line in Mouse byWnt10b Expression. Dev. Dyn. 229, 349–356. 10.1002/dvdy.10441 14745960

[B150] Wahba H. A. El-Hadaad H. A. (2015). Current Approaches in Treatment of Triple-Negative Breast Cancer. Cancer Biol. Med. 12, 106–116. 10.7497/j.issn.2095-3941.2015.0030 26175926 PMC4493381

[B151] Waks A. G. Winer E. P. (2019). Breast Cancer Treatment. Jama 321, 288–300. 10.1001/jama.2018.19323 30667505

[B152] Wang J. Wakeman T. P. Lathia J. D. Hjelmeland A. B. Wang X.-F. White R. R. (2010). Notch Promotes Radioresistance of Glioma Stem Cells. Stem Cells 28, 17–28. 10.1002/STEM.261 19921751 PMC2825687

[B153] Wang W. Li M. Ponnusamy S. Chi Y. Xue J. Fahmy B. (2020). ABL1-dependent OTULIN Phosphorylation Promotes Genotoxic Wnt/β-Catenin Activation to Enhance Drug Resistance in Breast Cancers. Nat. Commun. 11, 1–15. 10.1038/s41467-020-17770-9 32770022 PMC7414915

[B154] Watson R. L. Spalding A. C. Zielske S. P. Morgan M. Kim A. C. Bommer G. T. (2010). GSK3β and β-Catenin Modulate Radiation Cytotoxicity in Pancreatic Cancer. Neoplasia 12, 357–365. 10.1593/neo.92112 20454507 PMC2864473

[B155] Weekes C. Berlin J. Lenz H.-J. O'Neil B. Messersmith W. Cohen S. (2016). Phase 1b Study of WNT Inhibitor Ipafricept (IPA, Decoy Receptor for WNT Ligands) with Nab-Paclitaxel (Nab-P) and Gemcitabine (G) in Patients (Pts) with Previously Untreated Stage IV Pancreatic Cancer (PC). Ann. Oncol. 27, vi117. 10.1093/annonc/mdw368.10

[B156] Wei B. Cao J. Tian J.-H. Yu C.-Y. Huang Q. Yu J.-J. (2021). Mortalin Maintains Breast Cancer Stem Cells Stemness via Activation of Wnt/GSK3β/β-Catenin Signaling Pathway. Am. J. Cancer Res. 11 (6), 2696–2716. 34249423 PMC8263651

[B157] Wend P. Runke S. Wend K. Anchondo B. Yesayan M. Jardon M. (2013). WNT10B/β‐catenin Signalling Induces HMGA2 and Proliferation in Metastatic Triple‐negative Breast Cancer. EMBO Mol. Med. 5, 264–279. 10.1002/emmm.201201320 23307470 PMC3569642

[B158] Wissmann C. Wild P. J. Kaiser S. Roepcke S. Stoehr R. Woenckhaus M. (2003). WIF1, a Component of the Wnt Pathway, Is Down-Regulated in Prostate, Breast, Lung, and Bladder Cancer. J. Pathol. 201, 204–212. 10.1002/path.1449 14517837

[B159] Wu Y. Ginther C. Kim J. Mosher N. Chung S. Slamon D. (2012). Expression of Wnt3 Activates Wnt/β-Catenin Pathway and Promotes EMT-like Phenotype in Trastuzumab-Resistant HER2-Overexpressing Breast Cancer Cells. Mol. Cancer Res. 10, 1597–1606. 10.1158/1541-7786.MCR-12-0155-T 23071104 PMC3732195

[B160] Xie T. Jiang C. Dai T. Xu R. Zhou X. Su X. (2019). Knockdown of XB130 Restrains Cancer Stem Cell‐like Phenotype through Inhibition of Wnt/β‐Catenin Signaling in Breast Cancer. Mol. Carcinog. 58, 1832–1845. 10.1002/mc.23071 31219645

[B161] Xie W. Zhao H. Wang F. Wang Y. He Y. Wang T. (2021). A Novel Humanized Frizzled-7-Targeting Antibody Enhances Antitumor Effects of Bevacizumab against Triple-Negative Breast Cancer via Blocking Wnt/β-Catenin Signaling Pathway. J. Exp. Clin. Cancer Res. 40, 30. 10.1186/s13046-020-01800-x 33436039 PMC7802198

[B162] Xu J. Prosperi J. R. Choudhury N. Olopade O. I. Goss K. H. (2015). β-Catenin Is Required for the Tumorigenic Behavior of Triple-Negative Breast Cancer Cells. PLoS ONE 10, e0117097–11. 10.1371/journal.pone.0117097 25658419 PMC4319896

[B163] Xu X. Zhang M. Xu F. Jiang S. (2020). Wnt Signaling in Breast Cancer: Biological Mechanisms, Challenges and Opportunities. Mol. Cancer 19, 165. 10.1186/s12943-020-01276-5 33234169 PMC7686704

[B164] Yamaguchi H. Chang S.-S. Hsu J. L. Hung M.-C. (2014). Signaling Cross-Talk in the Resistance to HER Family Receptor Targeted Therapy. Oncogene 33, 1073–1081. 10.1038/ONC.2013.74 23542173 PMC3874419

[B165] Yamaguchi N. Oyama T. Ito E. Satoh H. Azuma S. Hayashi M. (2008). NOTCH3 Signaling Pathway Plays Crucial Roles in the Proliferation of ErbB2-Negative Human Breast Cancer Cells. Cancer Res. 68, 1881–1888. 10.1158/0008-5472.CAN-07-1597 18339869

[B166] Yan Y. Liu F. Han L. Zhao L. Chen J. Olopade O. I. (2018). HIF-2α Promotes Conversion to a Stem Cell Phenotype and Induces Chemoresistance in Breast Cancer Cells by Activating Wnt and Notch Pathways. J. Exp. Clin. Cancer Res. 37, 256. 10.1186/s13046-018-0925-x 30340507 PMC6194720

[B167] Yang L. Tang H. Kong Y. Xie X. Chen J. Song C. (2015). LGR5 Promotes Breast Cancer Progression and Maintains Stem-like Cells through Activation of Wnt/β-Catenin Signaling. Stem Cells 33, 2913–2924. 10.1002/stem.2083 26086949

[B168] Yang L. Wu X. Wang Y. Zhang K. Wu J. Yuan Y.-C. (2011). FZD7 Has a Critical Role in Cell Proliferation in Triple Negative Breast Cancer. Oncogene 30, 4437–4446. 10.1038/onc.2011.145 21532620

[B169] Yu Q. Verheyen E. Zeng Y. (2016). Mammary Development and Breast Cancer: A Wnt Perspective. Cancers 8, 65–26. 10.3390/cancers8070065 PMC4963807 27420097

[B170] Zeng Y. A. Nusse R. (2010). Wnt Proteins Are Self-Renewal Factors for Mammary Stem Cells and Promote Their Long-Term Expansion in Culture. Cell Stem Cell 6, 568–577. 10.1016/j.stem.2010.03.020 20569694 PMC2917779

[B171] Zhan T. Rindtorff N. Boutros M. (2017). Wnt Signaling in Cancer. Oncogene 36, 1461–1473. 10.1038/onc.2016.304 27617575 PMC5357762

[B172] Zhang H. Zhang X. Wu X. Li W. Su P. Cheng H. (2012). Interference of Frizzled 1 (FZD1) Reverses Multidrug Resistance in Breast Cancer Cells through the Wnt/β-Catenin Pathway. Cancer Lett. 323, 106–113. 10.1016/j.canlet.2012.03.039 22484497

[B173] Zhang X. Cheong S.-M. Amado N. G. Reis A. H. MacDonald B. T. Zebisch M. (2015). Notum Is Required for Neural and Head Induction via Wnt Deacylation, Oxidation, and Inactivation. Dev. Cell 32, 719–730. 10.1016/j.devcel.2015.02.014 25771893 PMC4375027

[B174] Zhang Y. Wang X. (2020). Targeting the Wnt/β-Catenin Signaling Pathway in Cancer. J. Hematol. Oncol. 13, 1–16. 10.1186/s13045-020-00990-3 33276800 PMC7716495

[B175] Zhang Z.-M. Wu J.-F. Luo Q.-C. Liu Q.-F. Wu Q.-W. Ye G.-D. (2016). Pygo2 Activates MDR1 Expression and Mediates Chemoresistance in Breast Cancer via the Wnt/β-Catenin Pathway. Oncogene 35, 4787–4797. 10.1038/onc.2016.10 26876203

[B176] Zheng H. Bae Y. Kasimir-Bauer S. Tang R. Chen J. Ren G. (2017). Therapeutic Antibody Targeting Tumor- and Osteoblastic Niche-Derived Jagged1 Sensitizes Bone Metastasis to Chemotherapy. Cancer Cell 32, 731–747. e6. 10.1016/j.ccell.2017.11.002 29232552 PMC5729937

[B177] Zhong Z. Virshup D. M. (2020). Wnt Signaling and Drug Resistance in Cancer. Mol. Pharmacol. 97, 72–89. 10.1124/MOL.119.117978 31787618

[B178] Zhu L. Pan R. Zhou D. Ye G. Tan W. (2019). BCL11A Enhances Stemness and Promotes Progression by Activating Wnt/β-Catenin Signaling in Breast Cancer. Cancer Manag. Res. 11, 2997–3007. 10.2147/CMAR.S199368 31114347 PMC6489585

